# Capturing fine-scale coral dynamics with a metacommunity modelling framework

**DOI:** 10.1038/s41598-024-73464-y

**Published:** 2024-10-21

**Authors:** Anna K. Cresswell, Vanessa Haller-Bull, Manuel Gonzalez-Rivero, James P. Gilmour, Yves-Marie Bozec, Diego R. Barneche, Barbara Robson, Kenneth R. N. Anthony, Christopher Doropoulos, Chris Roelfsema, Mitchell Lyons, Peter J. Mumby, Scott Condie, Veronique Lago, Juan-Carlos Ortiz

**Affiliations:** 1 Australian Institute of Marine Science, Perth, WA 6009 Australia; 2 Oceans Institute, University of Western Australia, Perth, WA 6009 Australia; 3 Australian Institute of Marine Science, Townsville, QLD 4810 Australia; 4 School of the Environment, The University of Queensland, Brisbane, QLD 4072 Australia; 5 CSIRO Environment, St. Lucia, QLD 4067 Australia; 6 University of New South Wales, Sydney, NSW 2052 Australia; 7 CSIRO Environment, Hobart, TAS 7001 Australia; 8 AIMS@JCU (aims@jcu.edu.au), Townsville, Queensland Australia

**Keywords:** Population dynamics, Ecological modelling, Community ecology, Ecosystem ecology, Restoration ecology

## Abstract

Natural systems exhibit high spatial variability across multiple scales. Models that can capture ecosystem dynamics across space and time by explicitly incorporating major biological mechanisms are crucial, both for management and for ecological insight. In the case of coral reef systems, much focus has been on modelling variability between reefs, despite substantial variability also existing within reefs. We developed *C*~*scape,* a coral metacommunity modelling framework that integrates the demography of corals with population-level responses to physical and environmental spatial layers, to facilitate spatiotemporal predictions of coral dynamics across reefs at fine (100s of metres to kilometres) scales. We used satellite-derived habitat maps to modulate community growth spatially, as a proxy for the many interacting physical and environmental factors—e.g., depth, light, wave exposure, temperature, and substrate type—that drive within-reef variability in coral demography. With a case study from the Great Barrier Reef, we demonstrate the model’s capability for producing hindcasts of coral cover dynamics and show that overlooking within-reef variability may lead to misleading conclusions about metacommunity dynamics. *C*~*scape* provides a valuable framework for exploring a range of management and restoration scenarios at relevant spatial scales.

## Introduction

Complex ecosystems like coral reefs are governed by processes operating across a range of spatial and temporal scales. Predicting the future of coral reefs to inform management actions requires a strong understanding of reef functioning across these scales. A considerable body of literature establishes the importance of variation between reefs, at scales from 10 to 1000s of km, and elucidates how physical and environmental factors influence coral composition, functioning and temporal dynamics across these large scales^1–7^. Similarly, substantial variation also exists at smaller scales within reefs, most obviously manifesting as differences between reef zones (i.e. reef slope, flat or lagoon,^8–10^), but also varying locally at finer resolutions, from meters to kilometres. Local physical and environmental factors, such as depth, light, wave exposure, water circulation, temperature, and substrate type, drive this variability within reefs^11–14^. Understanding within-reef variability is crucial, as coral populations in different parts of a reef may have varying influences on the overall growth, survival, and recovery of a reef^10^. Assuming one part of a reef is indicative of the entire reef’s functioning, or calculating an average of several sites without accounting for the distribution of coral populations, can lead to inaccurate conclusions about the overall health and performance of a reef^15^. Furthermore, in the context of applied conservation, some locations may provide more optimal restoration or management areas^15–17^. However, obtaining insights into within-reef variability is hampered by limited field data and modelling at scales that capture dynamics across the heterogenous seascape of a reef.

Disturbances, such as cyclones, marine heatwaves, and outbreaks of coral-eating predators are major structuring forces on coral reefs, often causing drastic declines in coral populations^18,19^. However, the stress experienced during acute disturbances can vary spatially across a reef due to the localised physical and environmental conditions^20–23^. For example, shallower sites with low water circulation often experience greater stress during marine heatwaves and therefore higher coral mortality^22,23^. Other patches of a reef, such as those with high water circulation or those at depth, may act as ‘refuges’ and subsequently contribute disproportionately to reef recovery (e.g.,^6,24^). Considering coral demography and larval connectivity^25^ across metapopulations is therefore essential to understand within-reef variability and test concepts such as recovery via remnant refugia populations.

Modelling coral demographic mechanisms—growth, survival, reproduction and larvae movement across a reef^1,26–28^—offers many opportunities for advancing coral reef research^26,28–31^, particularly when seeking to predict how corals will respond to unprecedented disturbance regimes. Integral Projection Models (IPMs) use empirical information on an individual’s demographic rates to estimate population level statistics^32,33^ and have been applied to many species across the animal and plant kingdoms^34–36^. Recently, the expanded application of IPMs from single populations to metapopulations has gained momentum^37,38^. Metapopulation modelling using IPMs offers potential to mechanistically investigate how changes in coral individuals may propagate to populations and metapopulations across space and time. There are, however, challenges with using projection models for long-term prediction as they were originally designed to study populations at equilibrium or at a particular point in time. Therefore they are not intrinsically constrained in their rates of growth, survival or fecundity by limiting factors^26,39,40^. For example, a population of sessile organisms, such as corals, cannot have indefinite positive population growth due to space limitation, and therefore population growth rate needs to be modulated by available space^15,40–42^. Furthermore, IPMs require large amounts of demographic data, particularly when trying to represent environmental gradients, although there are methods to circumvent data gaps^32,43^.

Population dynamics are often modelled using logistic ordinary differential equations, which have a population growth rate parameter and are limited by the availability of a critical resource, such as suitable habitat or food availability^42^. This differential equation approach has been applied successfully across many marine ecosystems^42,44–47^, but key demographic processes occuring at the individual level are not captured directly, making it difficult to isolate how a particular process (e.g. fecundity, adult growth or recruitment), influences the overall population growth. If demographic information on growth, survival and reproduction is available from sampling coral individuals, the population growth rate from an IPM can be used in an ordinary differential equation describing population change with time^41^. Such population models can then be combined into a community model, by accounting for the common utilisation of the limiting resource (e.g., space) by the populations which make up the community^42^. This approach can be used to create a community model with a more mechanistic foundation to demography, while still capturing the influence of resource depletion as maximum population size is approached.

The distribution and abundance of corals across a reef is a product of multiple interacting physical and biological processes, which collectively constrain the maximum coral cover and the rate at which that upper threshold is reached^15,42^. Remotely sourced data accounting for variability in environmental, physical and biological parameters offers an option for defining this upper threshold^48,49^. Geomorphic and benthic habitat maps have been used to estimate the coral habitat on the Great Barrier Reef^50^ and this information could be used to modulate coral population growth as a function of available suitable habitat, as a proxy for the many processes that ultimately determine the distribution of corals and their demographic rates. These maps can also facilitate partitioning reefs into smaller sites of relatively homogenous physical and environmental conditions.

In this study, we built a metacommunity modelling framework that integrates the demographic rates of coral individuals with community-level responses to spatial and temporal environmental variation. We utilise high-resolution habitat maps to construct a spatially explicit seascape of coral populations within sites across reefs, with each site containing a simple community of two coral types. We use fine-scale larval connectivity modelling^25^ to describe connections among sites and create a ‘metacommunity’. Integral Projection Models, constructed using empirical data for a corymbose *Acropora* and a small sub-massive coral type, inform population growth in a logistic growth community model, which adjusts for reductions in growth as the maximum available coral habitat is approached. We compared two parameterisations for coral habitat: assuming a uniform distribution across the reef versus considering spatial variation informed by habitat maps. For the former, we assumed a maximum coral cover of 80%, based on assumptions in previous studies regarding this upper limit (e.g. 100%^4,45^, 50–90%^5^, or parameterisation from empirical data^15,51^). For the latter, we developed a methodology to derive a site-specific coral habitat parameter from the habitat maps to explore the potential for map-derived information to act as a proxy for processes influencing coral distribution and demogaphy across a reef. We tested both parameterisations with a case study of five reefs in the central/northern Great Barrier Reef. We used long-term monitoring data to assess whether the site-specific coral habitat parameterisation captured within-reef variability and allowed more accurate hindcasts of reef trajectories than assuming uniform coral habitat across reefs. We contextualize how within-reef variability may influence conclusions about average reef dynamics.

## Results

### *C*~*scape* model framework

**Fig. 1:**
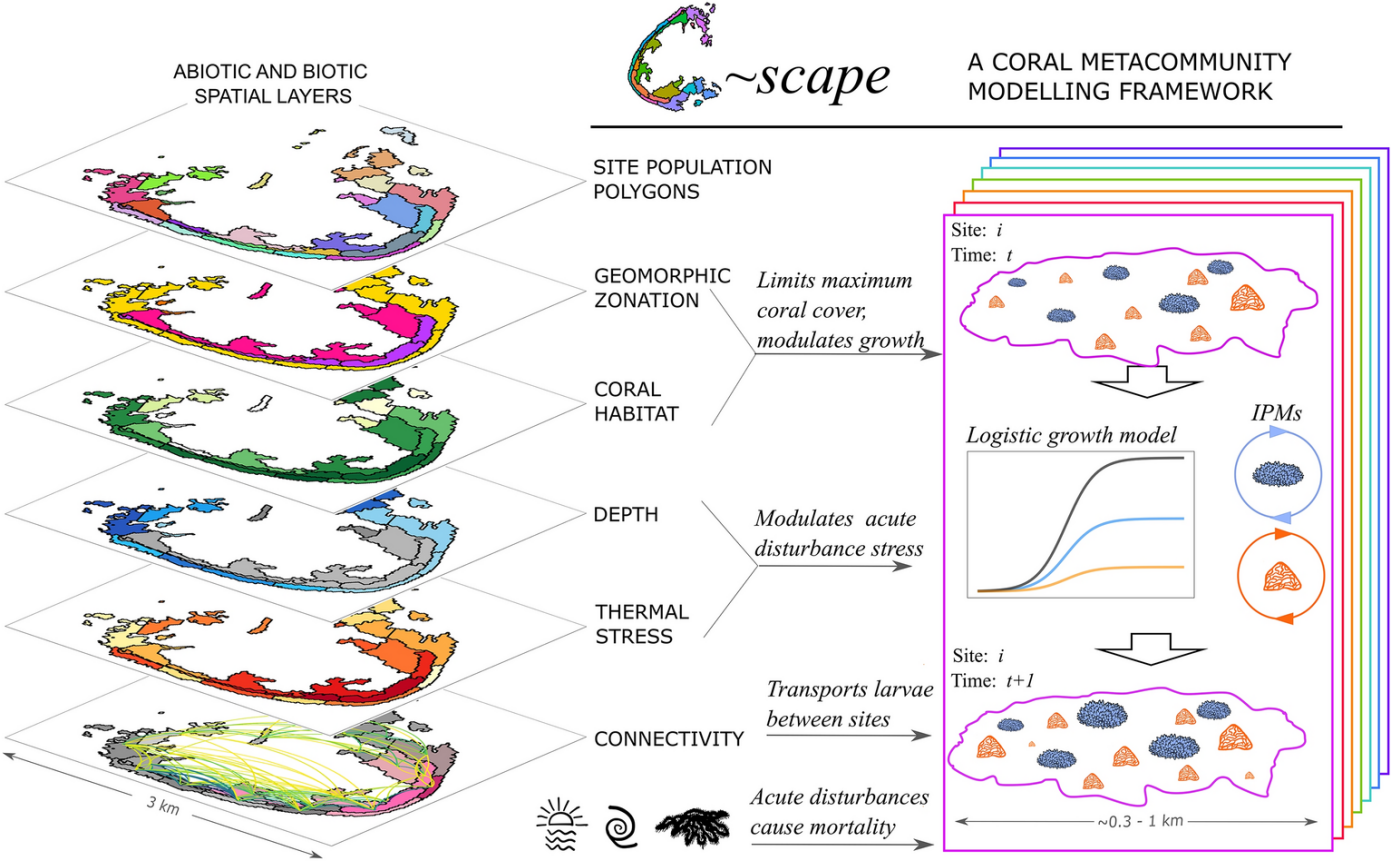
Schematic representation of the C~scape framework. C~scape is applied to a reef or cluster of reefs. The reef(s) are divided into spatial units for modelling: ‘sites’. A coral population for each of two distinct coral types—a corymbose *Acropora* and a small sub-massive coral—is discretely modelled at each site, with these populations connected by a connectivity matrix representing the probability of coral larvae moving between sites. The abundance and size of corals in each site are tracked. Sites have different depths, influencing the populations’ exposure to temperature stress. Information on thermal stress variability and connectivity between sites is required. Intrinsic population growth rates are determined for each coral type from Integral Projection Models (IPMs) that describe the coral life cycle, enabling the projection of changes in each population of corals over an annual period. The IPM contains discrete life states for eggs, larvae and settlers, as well as a continuous state across the size range of each coral type. Sites are assigned a ‘coral habitat’ value, which specifies the maximum percentage total coral cover in a site—this value modulates community growth as the maximum is approached. Acute disturbances—temperature stress, cyclones and crown-of-thorns starfish outbreaks—may affect coral populations by killing individuals in any given year.

The *C*~*scape* modelling framework (Fig. 1) combines coral demography with the major processes that vary spatially within reefs and influence coral population growth, to project coral metacommunity dynamics (Fig. 3).

### A spatially explicit seascape

**Fig. 2:**
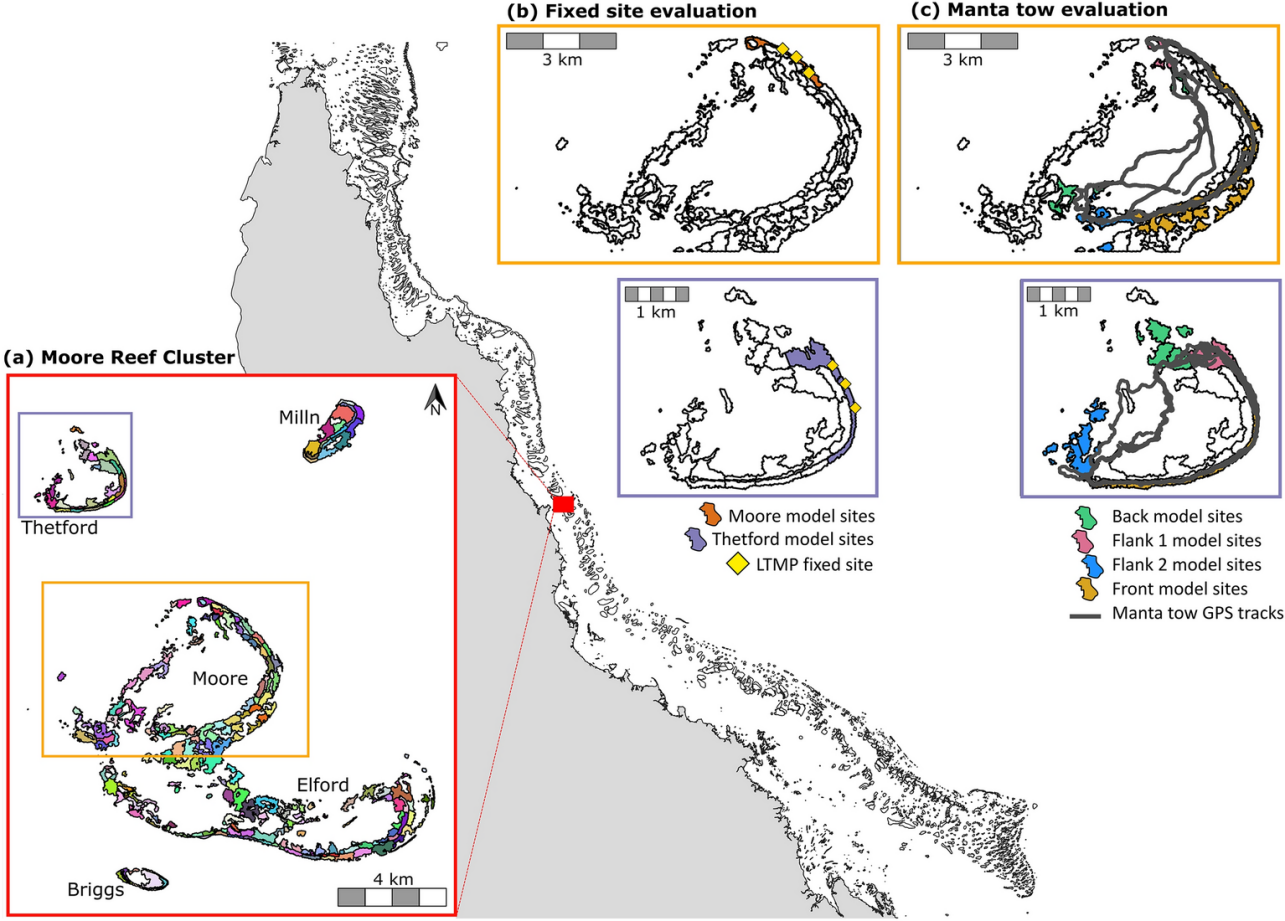
The northeast coast of Australia with the outlines of reefs on the Great Barrier Reef shown in grey (sourced from Great Barrier Reef Marine Park Authority Geohub) **a** The ‘Moore Reef Cluster’ case study, showing the reefs broken into sites (colours indicating distinct sites). Sites were delineated using a geomorphic habitat map described in^50^, also sourced from the Reef Authority Geohub. Each of the five reefs is labelled, and Moore reef and Thetford reef are delineated by rectangular outlines to indicate they are AIMS long-term monitoring (LTMP) sites with data available for model evaluation. The LTMP locations, and the overlapping model sites selected for comparative analyses, are shown in **b** for the fixed-position photo transect dataset and **c** for the manta tow dataset. In **b** yellow points show the location of the three fixed-position transects at each of the two reefs. In **c** the manta tow tracks are shown in grey. The model sites underlying these tracks, from which model data were compared to empirical data, are one of four colours representing the four manta tow sectors—back reef, flank 1, flank 2 and reef front—or white, meaning they were modelled but not included in comparison with LTMP data. Figure was created using R Version 4.3.0 using shapefile and raster inputs from the Reef Authority Geohub https://geohub-gbrmpa.hub.arcgis.com/.

Geomorphic habitat maps were used to partition a set of five reefs, the ‘Moore Reef Cluster’, offshore from Cairns on the central/ northern Great Barrier Reef, into a heterogenous seascape composed of 213 sites (Fig. 2), each with maximum longest axis of ~ 1 km. A simple coral community (composed of two distinct coral types—a corymbose *Acropora* and a small sub-massive coral—chosen to represent functionally distinct and common corals on the Great Barrier Reef) was modelled within each site. Two types of data from the AIMS long-term monitoring program (LTMP)^52^ allowed for comparison of the modelled total coral cover trajectories with those from fixed-position transects and from manta tow surveys at model sites overlapping with LTMP data (Fig. 2, see more detail in Methods).

### Spatiotemporal coral dynamics

**Fig. 3:**
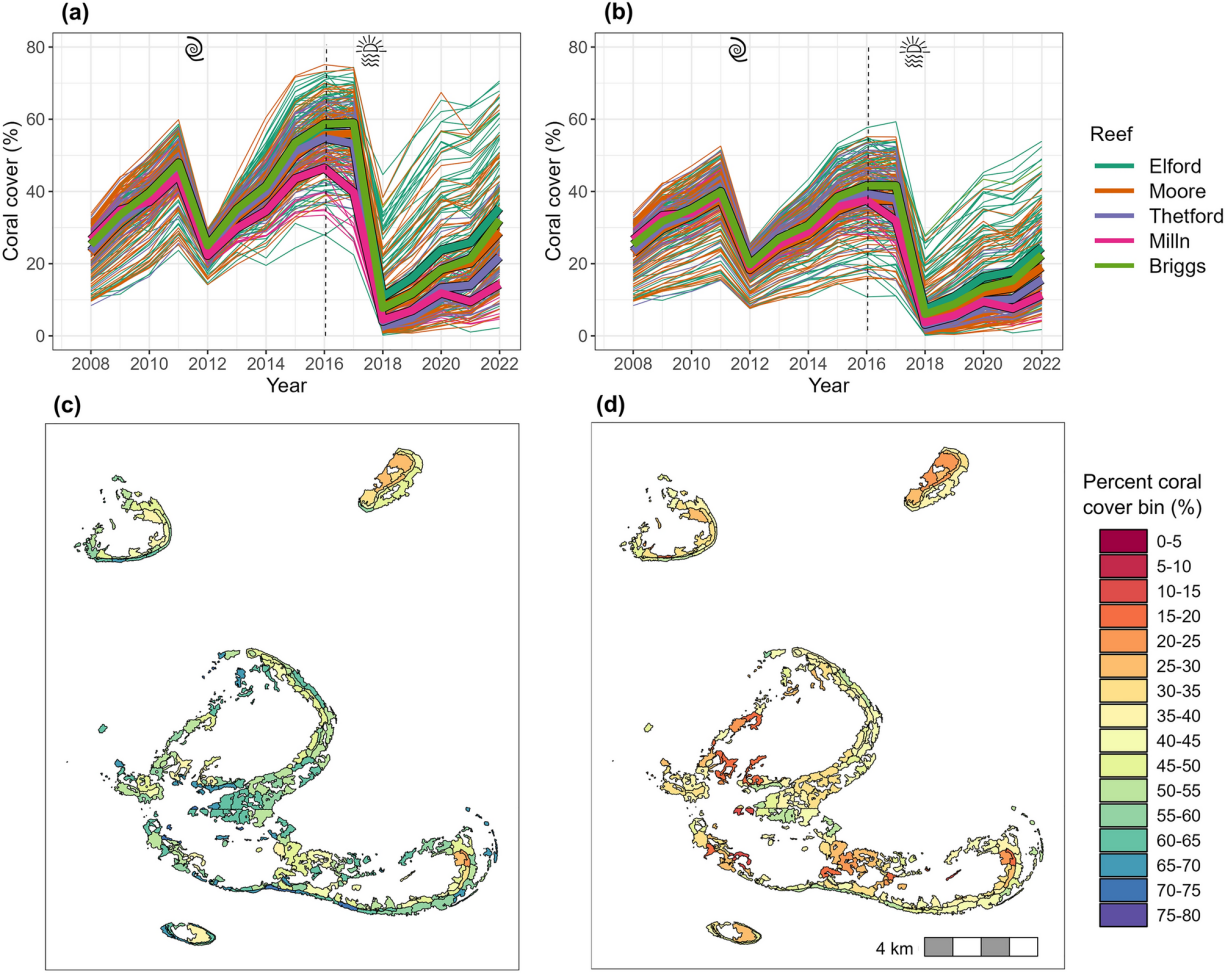
**a**, **b** Trajectories in coral cover between 2008 and 2022 where the model was informed by **a** an assumption of uniform coral habitat availability of 80% across the reef cluster and **b** site-specific coral habitat derived from benthic habitat maps. Each narrow line represents a site, with colour indicating one of five reefs in the cluster. Thick coloured lines bordered in black show the site average for each reef. **c** and **d** show the spatial patterns in coral cover in 2016 for the uniform and site-specific coral habitat availability scenarios, respectively.

We hindcasted dynamic coral cover trajectories for the 213 sites within the Moore Reef Cluster. The impacts of acute disturbances were evident as sharp declines in coral cover between 2011–2012 (cyclone) and between 2017–2018 (temperature stress), followed by recovery (Fig. 3a,b). We attribute spatial variation in these dynamics to the interactions between population growth, disturbance, connectivity, temperature stress, and fine-scale variation in coral habitat (Fig. 3c,d).

In comparing the model hindcasts for the two parameterisations of coral habitat (uniform value of 80% cover for all sites, versus the benthic map derived site-specific values, Fig. S2), we found higher maximum coral covers and faster recovery rates (as time-averaged annual change in coral cover) when using the uniform parameterisation, compared to the site-specific parameterisation (Fig. 3a versus b).

Variation in coral cover between modelled sites across the reef cluster was evident, with site coral cover differing by more than 40% between sites when the site-specific coral habitat parameterisation was used, particularly several years following disturbance (Fig. 3b). Variation among reefs was also present in both coral habitat parameterisations (with less variation under the uniform parameterisation), but this was far less than the variation between sites (Fig. 3a,b). The model projected the coral cover at each site in each year allowing visualisation across a spatially explicit map of sites. Figure 3c, d provides an example of the spatial distribution of coral cover at sites across the reef in 2016, when the coral had mostly recovered from the cyclone disturbance in 2011.

### Reproducing observed coral dynamics

**Fig. 4:**
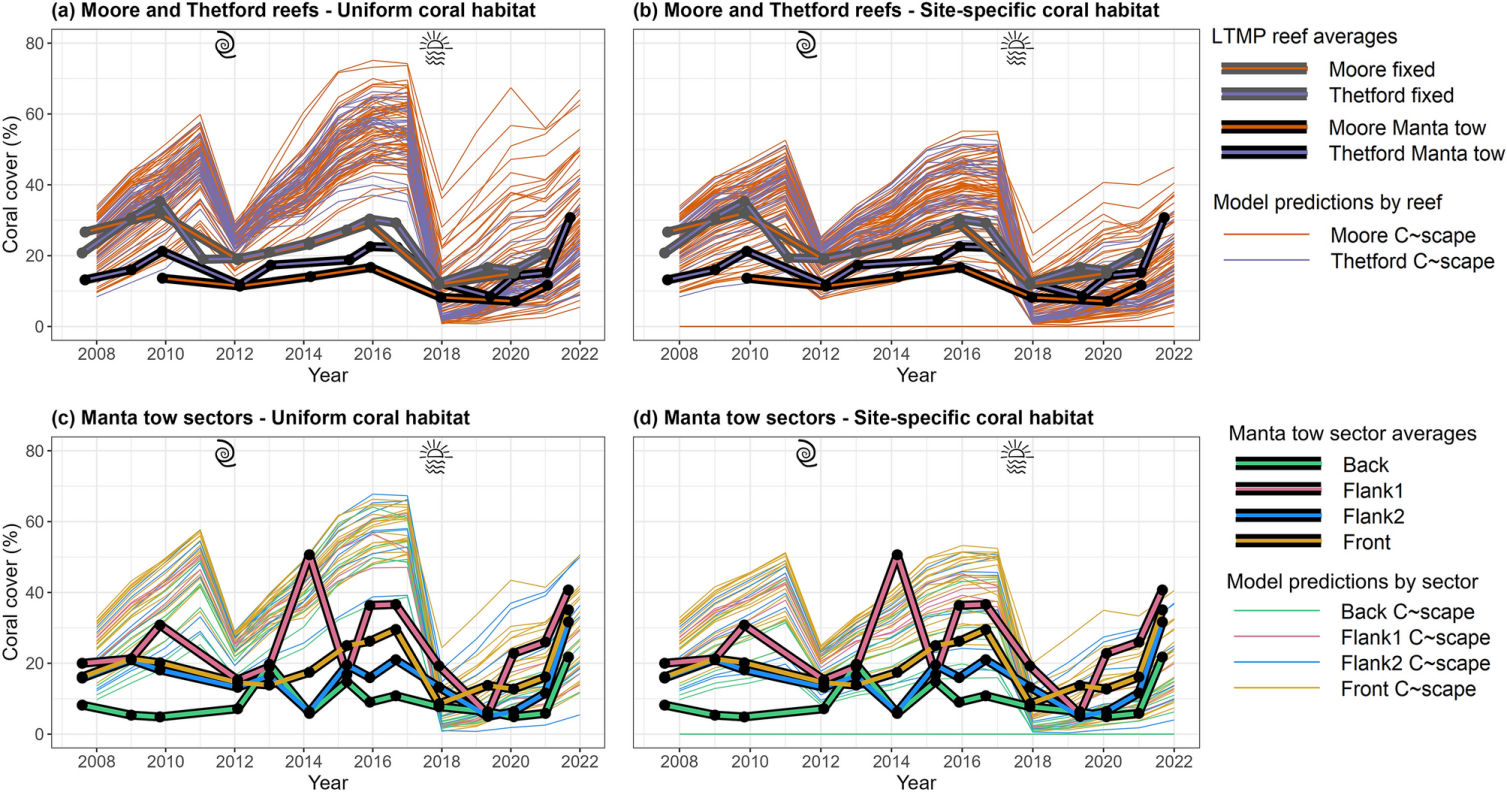
Modelled and observed coral cover dynamics at Moore and Thetford reefs. . Narrow lines show the trajectories of different modelled sites with colour indicating Moore or Thetford reefs in **a** and **b** and reef sector in **c** and **d**. Thick lines in **a** and **b** show the mean trajectories observed in the LTMP data for the two reefs, with bordering in grey showing the fixed position photo transects and bordering in black showing the manta tow data. Thick lines in **c** and **d** show the mean trajectories of the manta tow data broken into the four sectors and averaged across the two reefs. Variability associated with the empirical data can be viewed in Fig. 7. Left panels show the model output under the spatially uniform coral habitat parameterisation, while right panels show the modelled trajectories when coral habitat was parameterised to be site-specific using benthic habitat maps.

**Fig. 5:**
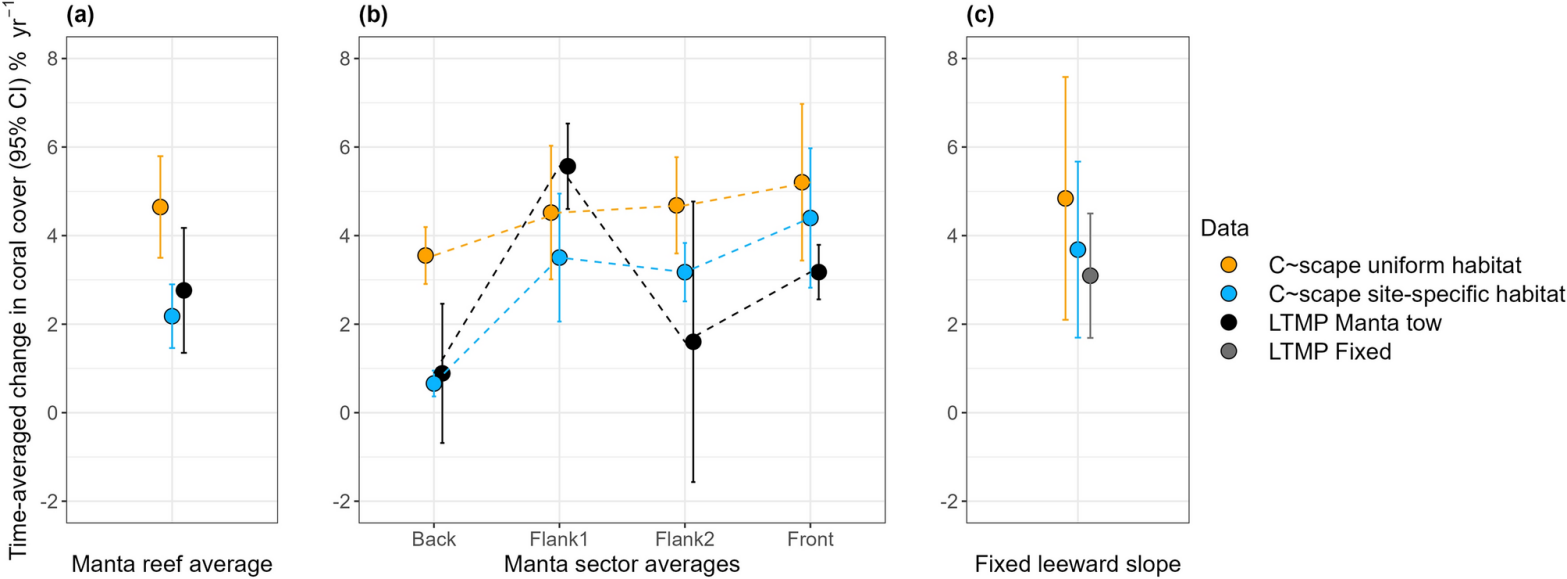
Time-averaged annual change in coral cover in the absence of acute disturbance for the growth windows shown in Fig. 7. **a** shows the predicted time-averaged annual change in coral cover for all sites underlying the manta tows, averaged across all growth windows, informed by the two different coral habitat parameterisations (uniform and site-specific), and the average time-averaged annual change across all manta tow observations. **b** shows the time-averaged annual change in coral cover broken down into the four different manta tow sectors. The dotted lines are included to aid visual interpretation of the pattern of variation between sectors. **c** shows the time-averaged annual change in coral cover at the LTMP fixed sites compared to the predicted time-averaged annual change in coral cover for the six polygons directly underlying these fixed sites at Moore and Thetford reefs.

Using the site-specific coral habitat parameterisation improved the match between modelled trajectories and the LTMP observations from both fixed-position photo transects and manta tow surveys (Fig. 4a,b). While the overlap in the model predictions and the observed LTMP trajectories was greater when the site-specific coral habitat parameterisation was used, the LTMP trajectories, in particular the manta tow data, had high variability between years, partly due to this being a rapid survey method that does not sample the exact same parts of reef each year, thus complicating comparisons (Fig. 4c,d, Fig. 7). Examining the time-averaged annual change in coral cover in the absence of acute disturbances therefore allowed a more quantitative comparison (Figs. 5 and 7).

### Time-averaged annual change in coral cover

According to the manta tow data, the time-averaged annual change in coral cover across Moore and Thetford reefs in the absence of acute disturbance was 2.76 ± 1.41% coral cover yr^-1^. The time-averaged annual change in coral cover predicted by *C*~*scape* for sites underlying the manta tows when parameterised with site-specific coral habitat was approximately 20% slower than the observed manta tow rate at 2.24 ± 0.72% yr^-1^ and with the confidence interval overlapping the manta tow observations (Fig. 5a). In contrast, under the parameterisation of uniform coral habitat, the time-averaged annual change was more than 1.6 times faster, at 4.49 ± 1.20% yr^-1^, with less overlap in the confidence intervals (Fig. 5a).

According to the fixed position transects on the leeward slope, the time-averaged annual change in coral cover was 3.09 ± 1.41% yr^-1^ in the absence of acute disturbance. The model predicted a slightly higher rate at 3.78 ± 2.04% yr^-1^ when parameterised with the site-specific coral habitat values, and higher again when uniform coral habitat was assumed (4.84 ± 2.74% yr^-1^, Fig. 5c).

The time-averaged annual change in coral cover for modelled hindcasts using the uniform coral habitat parameterisation showed some variation in rate between the different manta tow sectors of the reef (Fig. 5b), with the Front sector having a slightly faster rate than Flank 1 and 2, which in turn had a faster rate than the Back sector. These differences are not driven by the site-specific coral habitat parameter, given this is uniform across the reef, and can therefore be assumed to be driven by larval connectivity or variation in post-disturbance coral cover at each site due to temperature stress variability, and warrants further investigation in future work.

Differences between manta tow sectors were more pronounced when site-specific coral habitat values were assigned from the benthic habitat maps and the rate of change in coral cover matched more closely to the empirical data in this case. *C*~*scape* predicted fastest recovery rates in the Front and Flank 1 sectors, while Flank 2 had a slightly lower rate and recovery rate in the Back reef sectors was the lowest. The annual change in coral cover in the Back sector matched closely between empirical and modelled data, while *C*~*scape* somewhat overestimated recovery rate on the Flank 2 and Front sectors but underestimated on Flank 1. When comparing the time-averaged annual change in coral cover for LTMP fixed-position photo transects on the leeward slope of Moore and Thetford reefs, we found that *C*~*scape* overestimated the annual change in cover in this location, but to a lesser extent when using the site-specific habitat parameterisation.

**Fig. 6:**
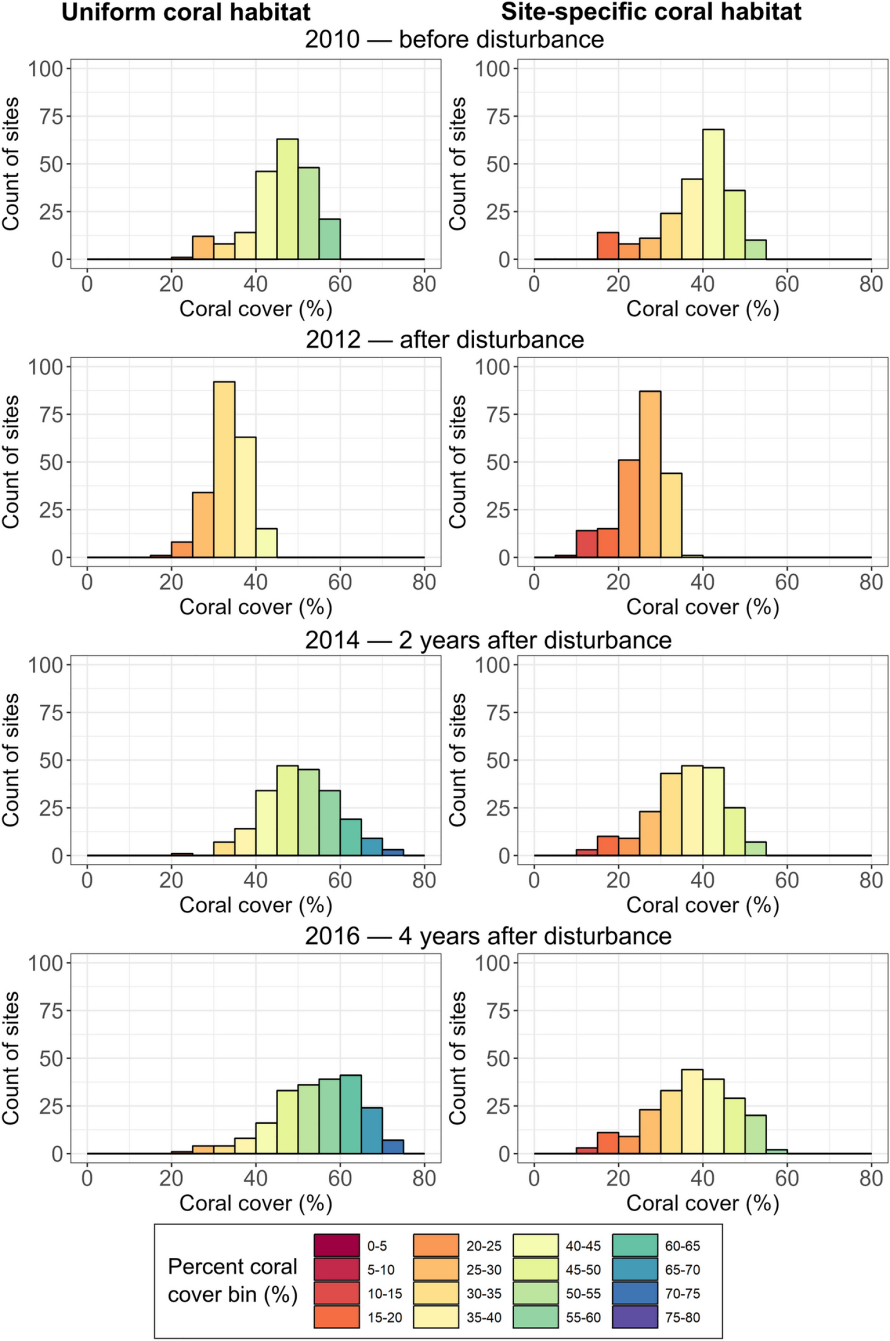
Histograms showing coral cover distribution across sites in the Moore Reef Cluster in four different years: 2010 (prior to disturbance), 2012 (the year after disturbance), 2014 (~ 2 years after disturbance), 2016 (~ 4 years after disturbance, also see Fig. 3c,d). Left panels show the distribution of coral cover when the coral habitat is assumed to be uniform across all sites with a value of maximum 80% coral cover. Right panels show the distributions when coral habitat was site-specific and informed from the benthic habitat maps.

*C*~*scape* produced outputs at a finer spatial scales than captured in the available empirical data. Therefore, these outputs cannot be validated at the site scale, but it is nonetheless valuable to examine the model predictions of within-reef variability. Variability between sites was temporally dynamic and was notably influenced by disturbance. Figure 6 summarises the coral cover across the *C*~*scape* sites at different times relative to disturbance. Disturbances caused a reduction in the variation in coral cover and a shift in the distribution to lower covers. The greatest variation within the Moore Reef Cluster was realised when the model was parameterised with the site-specific coral habitat values at the longest time since disturbance, ~ 4 years following the cyclone event in 2011.

As well as influencing population growth rate and maximum coral covers, the use of the site-specific parameterisation also had implications for the estimated area of coral that was projected across the modelled reef cluster. In the Moore Reef Cluster, the total modelled area, i.e., the sum of all site polygon areas, was 2259 hectares. When we assumed uniform coral habitat of 80% across the sites, the total possible live coral area would be 1807 ha, whereas informing the maximum coral habitat for each site based on the benthic habitat maps gave a smaller total area of 1207 ha.

## Discussion

We developed a coral metacommunity modelling framework, *C*~*scape*, with the goal of capturing fine-scale variation in coral habitat, acute disturbances, and larval connectivity, and the consequences for coral dynamics within and between reefs. We produced coral community dynamics for 213 discretely modelled sites, linked by larval connectivity across a case study of five reefs. By accounting for fine-scale variation in coral habitat suitability, heat stress, and connectivity, we hindcasted projections of coral populations over more than a decade (2008–2022), including capturing impacts from a coral bleaching event and a cyclone. The model framework includes many processes, but in this study, we focused on investigating whether incorporating a site-specific parameter for the suitable coral habitat could be used to spatially modulate a general community growth model and improve the ability to hindcast spatiotemporal dynamics. We built the model framework with a demographic foundation, by using Integral Projection Models (IPMs) to determine the intrinsic population growth rates for two coral types composing a simple coral community. While the most mechanistic approach to incorporating variation in demographic rates due to environmental gradients would be to include covariates such as depth, temperature, wave exposure and space availability into the statistics which underlie the IPMs^29,43^, the amount of high resolution individual-based coral data that would be required to do this was prohibitive. We show that within the framework of a combined IPM-logistic growth community model, habitat maps can be used to parameterise coral habitat and act as a proxy for the processes structuring within-reef variability in coral demographics in the absence of more detailed information on the specific drivers.

Comparing model performance under parameterisations of uniform coral habitat versus spatially explicit coral habitat revealed several important differences in the resulting hindcast coral dynamics. Modelled coral cover dynamics and time-averaged changes in cover were more comparable to the empirical observations in the long-term monitoring data when the site-specific parameterisation was used. As anticipated, the use of site-specific coral habitat parameterisation led to greater variation between sites. This variability was temporally dynamic and notably influenced by disturbance events, suggesting that some of this variability emerged from the interactions between processes in the model, such as between the demographic processes and connectivity or temperature stress. For instance, there was generally more variability several years following disturbance than immediately after disturbance under the site-specific parameterisation, but in the case of uniform coral habitat, this was less pronounced as all sites approached the uniform maximum total coral cover.

Coral cover dynamics varied between the five reefs in the case study, but there was greater variability between sites than between reefs, offering further support for the importance of considering small scale (100s meters to kms) variability. Similarly, empirical studies have shown that differences in environmental gradients across sites within a reef can drive greater differences in ecological interactions than between reefs with similar environmental exposures^53^. Although site-level predictions of coral dynamics could not be evaluated due to an absence of monitoring data at such fine spatial scales, we were able to examine the performance of the two coral habitat parameterisations in four different sectors of Moore and Thetford reefs. Comparison between the time-averaged rates of change in coral cover from the model and the manta tow data produced overlapping confidence intervals between reef sectors when the model was parameterised with the site-specific coral habitat values. This shows that even amidst other complex interacting processes in the *C*~*scape* framework, the inclusion of a site-specific coral habitat parameter that modulates coral demographics can allow within-reef dynamics to be better distinguished at the reef sector level. While there may be other ways to parameterise and model this variation more mechanistically^43^, such as parameterising spatially heterogeneous coral demographics, this would require substantial amounts of data on coral vital rates across environmental gradients.

A version of the satellite-derived habitat maps that were used to calculate the site-specific coral habitat values are available for coral reefs globally^49,54,55^, as well as for other ecosystems^56–58^. Our findings suggest that such mapping products can be used to generate a metric and proxy for the many processes that vary spatially within reefs. This greatly increases the feasibility of incorporating within-reef variability into other coral reef modelling and analysis efforts, which might otherwise not consider coral habitat suitability explicitly or assume it to be uniform across a reef. The approach to utilising estimates of habitat suitability from habitat maps provides an avenue for better representation of fine scale variability in other ecosystems where the main ecosystem engineers are sessile organisms that compete for space and resources. In marine environments, this could include kelp and mangrove forests and seagrass meadows, or in terrestrial environments, rainforests and grasslands. The modelling framework allows for the integration of new demographic and environmental data, and improved modelling of disturbance and connectivity processes, as this becomes available, which would improve model accuracy and broaden the scope of questions that can be explored.

Predictive models aspire to distil complex systems into a more manageable set of general patterns and processes to enable hindcasts and forecasts grounded in ecological behaviour^59^. Increasing the spatial resolution of modelling efforts has costs in computation and complexity and therefore considering (i) whether a higher resolution model can accurately replicate observed patterns, and (ii) if there are explicit questions or applications of a higher resolution model that remain unanswerable at a coarser scale. In this study, we have provided comparative analysis with long-term monitoring data to show that the *C*~*scape* modelling framework can at least partially satisfy the first condition. Improvements can be made to individual processes in the model, such as the demographic parameterisations and the connectivity modelling, which would enhance the capacity to reproduce observed reef patterns. We have identified a series of critical questions and applications that are best addressed with models at the within-reef spatial scale, thus satisfying point (ii):

- *Extrapolating spatiotemporal dynamics:* Long-term monitoring data is essential for understanding and managing natural systems, but it is costly and therefore the spatial coverage and resolution that can be obtained alongside fine-scale temporal data is limited^60,61^. This means that it is often necessary to generalise and take one part of a reef that has been sampled as representative of the reef more widely. This underestimates within-reef variability, and likely contributes to communication challenges between scientists, natural management agencies and stakeholders. Field data could be interpreted in conjuction with high resolution within-reef models to extrapolate how measurements from one part of a reef represent other parts of the reef.
- *Ecological questions that cannot be answered at a larger scale:* Many questions in coral reef ecology require consideration of within-reef variability. For example, identifying fine scale refugia from disturbance requires measuring or modelling exposure to disturbance, alongside the potential for larvae dispersal between different locations within a reef. Similarly, identifying the relative importance and interactions between processes such as demography, connectivity and habitat suitability in structuring coral distributions across a reef requires modelling at a within-reef scale.
- *Fine-scale areas of interest for management*: Although many conservation goals are focused on whole-of-system functionality, small areas of natural systems can have particular importance for society or ecological importance and are therefore given special conservation attention. For example, even in the case of the Great Barrier Reef, a reef system ~2,300 km in length that requires whole-of-reef management^62^, maintaining ecosystem functioning in small highly utilised areas—for example sites that tourist operators depend on, sites with high cultural values for Traditional Owners, or areas that are critical for fishing activities—requires local targeted approaches^63,64^. Furthermore, zoning for use management, e.g., marine reserves, while often covering large scales in the full extent, requires finer-scale boundaries that often partition reefs into different zones^65^. Models that focus on within-reef variability offer opportunities to improve the identification of within-reef zoning boundaries to optimisee zoning .
- *Guided deployment of interventions and restoration:* In response to the overwhelming threat posed by climate change, multiple initiatives worldwide are exploring innovative interventions focused at either reducing the magnitude of acute thermal stress or aiding recovery through seeding or planting natural or genetically adapted corals^31,66,67^. However, logistical and economic constraints significantly limit the area that can be targeted with these interventions. Consequently, identifying the reef(s) that, if chosen for intervention, would generate the highest system-level benefit is crucial. Yet even once reefs are chosen, identifying areas within a reef to deploy an intervention with the goal of generating the greatest reef-level benefit is also essential, particularly because many of the proposed interventions are deployed at a site scale rather than reef-wide (median restoration size = 100 m^2^^17^). This motivates the need for fine-scale modelling that can identify the characteristics of sites that have the most potential to increase reef-wide benefits or inform the choice of deployment location e.g.,^16^.

There are various ways benthic habitat maps or other types of maps describing geomorphology and substrate type could be used to calculate the coral habitat parameter used in this study. We presented the methodology for one approach, which created a significant improvement in the predictive accuracy of the *C*~*scape* model. Further work could investigate ways to improve the calculation of suitable coral habitat, particularly as the spatial and taxonomic resolution and accuracy of these maps continues to improve.

## Conclusions

We investigated the relevance and implications of within-reef variability, introducing the *C*~*scape* framework as a tool for ecological investigations and coral reef management in the Anthropocene. We aimed to address gaps in existing modelling efforts by incorporating spatial scales, biological mechanisms and environmental processes critical for effective management and restoration in the twenty-first century. We integrated high-resolution seascape mapping with Integral Projection Models to capture key demographic processes with a logistic growth community model. We demonstrated some of the model capabilities, in particular illustrating the practicality of using high resolution habitat maps to investigate and model within-reef variation. The principle of combining newly available high-resolution spatial information (now accessible for coral reefs worldwide^54^) to downscale models can extend to other marine and terrestrial ecosystems where sessile organisms compete for a space-related resource. The model is relevant for a wide range of questions which were beyond the scope of the present study, including predicting of reef futures and investigating the relative importance and uncertainty surrounding the other processes contributing to within-reef variability—such as larval connectivity, depth, wave exposure, spatial heterogeneity in acute stressors and demography. The *C*~*scape* model framework lays a strong foundation to explore these questions and advance our understanding of coral reef dynamics.

## Materials and methods

### *C*~*scape* model framework

The *C*~*scape* modelling framework (Fig. 1) integrates coral demography with the major processes that vary spatially within reefs to project coral metacommunity dynamics.

Spatial units for modelling are delineated as a mosaic of ‘site’ polygons across a chosen set of reefs. When delineating these sites, the aim is to reasonably assume relatively homogenous environmental conditions within each site compared to variations between sites (Table 1). Coral populations are modelled separately within each site but are connected via the transport of coral larvae between sites, thus forming ‘metapopulations’.

**Table 1:** Terminology and description of spatial scales pertaining to the C~scape model framework and specifics regarding the Moore Reef Cluster case study.

Term	Approximate scale	Description	Moore Reef Cluster case study
	Spanning 0.1–1 km across site distance ~ 1–25 ha	Sites are spatial polygons which vary in shape and are constrained to be within one of four geomorphic zones. Each site is assumed to have relatively homogeneous physical and environmental conditions and a population of two coral types. Sites are comparable to ‘patches’ in landscape ecology^68^. The network of coral populations across all sites constitutes the ‘metapopulation.’ Two coral types are modelled within each site, constituting a simple ‘metacommunity’.	213 sites were simulated. The median diameter was 340 m when sites assumed circular.
	Spanning 1–100 km across reef distance ~ 100–1e6 ha	Reefs are relatively discrete and continuous areas of hard substrate. Reefs can be partitioned into geomorphic zones (e.g., slope, sheltered reef slope, crest and outer reef flat)^50^. Reefs vary in size and are divided into sites.	Reefs range from 1.5 to 10 km in maximum diameter with between 7 and 95 sites per reef.
	Spanning 10–100 km across reef cluster distance ~ 1000–1e6 ha	A reef cluster is a group of reefs within which coral larvae are assumed to be routinely exchanged. The boundaries of a reef cluster can be user-selected based on interest or a priori information on larval connectivity between reefs. The boundary delineates the domain for fine-scale biophysical and coral population modelling. Import of coral and crown-of-thorns starfish larvae to the reef cluster from surrounding reefs is parameterised from larger-scale reef system models.	We modelled the Moore Reef Cluster, which contained 5 reefs.
Reef system	Spanning 100–1000s km across reef system distance ~ 1e6–1e8 ha	A reef system can span across regional scales, up to 1000s of km, encompassing all reefs along a continental margin or surrounding islands or oceanic atolls. Within a reef system coral larvae may exchange incrementally or over evolutionary time frames.	The Great Barrier Reef.

Each population is modelled using a logistic ordinary differential equation adapted such that the intrinsic growth rate is substituted for the growth rate derived from Integral Projection Models (IPMs,^33^). The IPMs are based on the coral life cycle (see Fig. S6), with one representing each of two distinct coral types (a corymbose Acropora and a small sub-massive coral), and allow predictions of the annual probability of growth and survival, and the amount of reproductive material produced by a population of corals in a site. To account for changes in coral growth and survival due to limits on available suitable habitat, growth in the IPMs is modulated by a coral habitat parameter that defines the maximum total coral cover in a site (further detail in subsequent sections).

Annual environmental forcings that cause coral mortality, i.e., temperature stress, crown-of-thorns starfish outbreaks and cyclones are assigned to each site.

The development of IPMs, site delineation and the main model simulation are programmed in R (Version 4.3.0^69^).

### Site population polygons: spatial units for modelling

The geographic boundaries of reefs were determined from a publicly available^70^ geomorphic zonation map of the Great Barrier Reef that depicts the distribution of geomorphic features such as reef crest, slopes, reef flats and lagoons^50^ sourced from Great Barrier Reef Marine Park Authority Geohub https://geohub-gbrmpa.hub.arcgis.com/. The geomorphic zonation map extends to a depth of approximately 20 m, and this establishes the depth limit of our current modelling efforts.

It is well established that different geomorphic zones are formed by distinctive environmental conditions^10^. Consequently, coral community composition and dynamics tend to be more similar within geomorphic zones than between them. Following the recommendations of Kennedy et al.^49^, we only modelled the zones assumed to have predominantly hard substrate—Reef Slope, Reef Crest, Outer Reef Flat, and Sheltered Reef Slope—as areas where we would expect appropriate habitat and conditions for corals to grow (described in Table S1,^50^).

Geomorphic zones were further partitioned to account for other spatially varying processes (e.g., connectivity, temperature stress). We pixelated each geomorphic zone into hexagonal cells in R using the geospatial indexing system H3 in package h3^71^ at a resolution of 12 (approximately 20 m diameter, Fig. S1). A distance matrix based on Euclidean distances was then created and the cells were clustered using the base R function ‘hclust’ with method set as ‘complete’. In this way, hexagons within the same geomorphic zone and adjacent to each other were clustered together to create a site polygon that had a maximum longest diameter of ~ 1 km. Sites did not need to be continuous (i.e., they could consist of multiple smaller polygons) provided they were in the same geomorphic zone.

### Community growth model

To project coral community growth over annual time steps we used a logistic ordinary differential growth equation, which can be written as

1 $${C}_{i,t+1}={C}_{i,t}+{rD}_{i,t}{C}_{i,t}$$

where ${C}_{i,t+1}$ is the total coral cover at time *t* + *1* at site *i*, ${C}_{i,t}$ is the total coral cover at time *t* at site *i*, ${D}_{i,t}$ is a parameter accounting for density dependent changes to growth as the maximum potential coral cover is approached, and *r* is the intrinsic rate of community growth.

We considered populations of two distinct coral types, *ft,* to compose a simple coral community. Coral cover was determined from the coral population size structure, $$\overset{\lower0.5em\hbox{$\smash{\scriptscriptstyle\rightharpoonup}$}}{{N_{ft,i,n,t} }}$$, at each time step, for each site:

2 $$C_{i,t} = \mathop \sum \limits_{ft = 1}^{2} \mathop \sum \limits_{n = 1}^{100} \overset{\lower0.5em\hbox{$\smash{\scriptscriptstyle\rightharpoonup}$}}{{N_{ft,i,n,t} }} \times \overset{\lower0.5em\hbox{$\smash{\scriptscriptstyle\rightharpoonup}$}}{{a_{ft,n} }}$$

where $${a}_{ft,n}$$ is the colony area corresponding to the *n*th of 100 size classes for coral type *ft,* and $$\overset{\lower0.5em\hbox{$\smash{\scriptscriptstyle\rightharpoonup}$}}{{N_{{ft,{\text{i}},n,t}} }}$$ is a vector describing is the abundance of colonies of each functional type *ft*, in each size class *n,* at site *i*, at time *t.*

To describe the intrinsic rate of population growth, ${r}_{ft}$*,* for coral type *ft*, we modified the approach in Miller et al.^41^ and used an integral projection matrix to describe the probability of transitioning between size classes, such that

3 $${r}_{ft}=\left(IP{M}_{ft}-{I}_{ft}\right)$$

where $IP{M}_{ft}$ is the integral projection matrix, and ${I}_{ft}$ is the identity matrix (the diagonal of the IPM matrix that describes the probability of a coral colony staying the same size, see Miller et al.^41^) of coral type *ft*. By subtracting the identity matrix, what remains is the probabilities of growth, shrinkage and survival, such that the intrinsic rate of population growth describes how individuals change in size and what individuals are added/subtracted from the population.

To account for the effect of space limitation on coral community growth, density dependence, ${D}_{i,t}$ for site *i* at time *t*, was calculated as:

4 $${D}_{i,t}=\frac{{K}_{i}-{C}_{i,t}}{{K}_{i}}$$

where ${K}_{i}$ is the coral habitat at site *i*, and ${C}_{i,t}$ is the total coral cover at site *i* at time *t.*${K}_{i}$ is designed to act as a proxy for the many interacting physical and environmental factors—e.g., depth, light, wave exposure, temperature, and substrate type—that set the limit on the maximum amount of coral cover that can be obtained.

The final equation to project changes in each coral population *ft*, in terms of size structure, while accounting for coral habitat ${K}_{i}$, at site *i* at time *t,* is a discrete time-scalar logistic growth equation:

5 $$\overset{\lower0.5em\hbox{$\smash{\scriptscriptstyle\rightharpoonup}$}}{{N_{ft,i,t + 1} }} = \overset{\lower0.5em\hbox{$\smash{\scriptscriptstyle\rightharpoonup}$}}{{N_{ft,i,t} }} + r_{ft} D_{i,t} \overset{\lower0.5em\hbox{$\smash{\scriptscriptstyle\rightharpoonup}$}}{{N_{ft,i,t} }}$$

where $$\overset{\lower0.5em\hbox{$\smash{\scriptscriptstyle\rightharpoonup}$}}{{N_{ft,i,t + 1} }}$$ is a vector describing the number of colonies in each of the 100 size classes at *t* + *1* and three discrete life stages (eggs, larvae and settlers), and $$\overset{\lower0.5em\hbox{$\smash{\scriptscriptstyle\rightharpoonup}$}}{{N_{ft,i,t} }}$$ is a vector describing the same at time *t.*

The intrinsic growth rate of each population is multiplied by the community crowding function, such that population growth is reduced as the two populations fill the available space, ensuring they do not exceed the maximum coral habitat in a site, ${K}_{i}$.

### Integral Projection Models

The integral projection transition matrix, or kernel $k\left(y,x\right)$, is analogous to a projection matrix in matrix population modelling^72^. It represents all possible transitions from state $x$ (in year $t$) to state *y* (in year $t+1$), integrated across all states of $x$^73^:

6 $$k{\left(y,x\right)}=s\left(x\right)g{\left(x,y\right)}+f{\left(x,y\right)}$$

where $s(x)$ is survival of individuals in state $x$ from time t to t + 1, $g(x,y)$ is the growth of individuals from state $x$ to state $y$, $f(x,y)$ is the fecundity of individuals in state $x$ producing those in state $y$ at t + 1^29^.

We modelled three discrete states for corals (eggs, larvae and settlers), and a continuous state (coral size). Illustrated in Fig. S6, we model, or parameterise, the (i) growth and (ii) survival, (iii) fecundity (egg production), (iv) fertilisation (the number of eggs that are fertilised and become larvae), (vi) the survival and settlement probability of the larvae (handled separately to the IPM so that (v) connectivity modelling can be used to simulate the exchange of larvae among sites), and (vii) survival of settlers and growth to a 1-year old coral at which point corals enter the continuous state (Fig. S6, Table S1).

We fit regressions for each of growth, survival, and fecundity as a function of coral colony surface area (cm^2^) in a Bayesian framework following the approach of Elderd and Miller^74^ and Kayal et al.^75^ using the brms package in R^76^.

Colony growth was modelled by predicting coral colony planar area at time t + 1 as a function of coral colony area at time t. Colony survival was modelled by predicting the binary output of alive or dead at time t + 1 as a function of colony area at time t. For fecundity, we modelled the number of eggs produced by a coral colony as a function of colony area. We also fitted a model for the number of polyps per cm^2^ coral area as a function of coral size. By multiplying predictions from these models we estimated the number of eggs as a function of colony size (more detail available in Supplementary Information 1.1).

Modifying code provided in Kayal et al.^75^, we predicted from the regressions for growth, survival and fecundity for 100 coral size classes (spanning from 1 cm diameter to 90% of the largest coral size in the dataset) for each coral type. We converted these predictions into transition probability, matrices. The transition matrices describe the probability of transitioning among all 100 size classes and the three discrete life stages (see further detail in Supplementary Information 2).

### Coral types and data

IPMs require substantial amounts of data to describe the vital rates—growth, survival and fecundity—as a function of a continuous state (here, coral planar area) with robust statistical regressions. Similar to previous studies^77^, we included two distinct coral functional types in the model: ‘corymbose *Acropora’* and ‘small sub-massive' coral (data from *Goniastrea* spp.). While additional coral types would provide deeper insights into community dynamics, these two groups represent two major functional groups on the Great Barrier Reef with distinct traits: corymbose *Acropora* is a relatively fast-growing coral with lower average survival, and relatively high vulnerability to thermal and hydrodynamic stress; in contrast, sub-massive *Goniastrea* are typically slower growing, have high annual survival, and are more resistant to thermal and hydrodynamic stress.

Ideally data for these coral types would come from the location where *C*~*scape* is to be applied, but here our focus was to develop a robust framework and so we used the largest demography datasets available to us. To inform the growth and survival components of the IPMs for corymbose *Acropora*, datasets were utilised from Scott Reef (northwest Australia,^78^), Heron Island (Great Barrier Reef,^79^), and Moorea (French Polynesia,^75^). These datasets employed comparable data collection methods, involving the tagging, photographing with a scale, and bi-annual or annual monitoring of tagged coral colonies. Data for sub-massive *Goniastrea* was obtained from the same Scott Reef dataset as for corymbose *Acropora*. Colony diameter was consistently measured across the three datasets, so we used diameter and converted this to colony area by assuming the colonies were circular.

Information on the number of eggs per coral polyp and number of polyps per colony surface area were available for *Acropora millepora* in the Scott Reef dataset. Additional parameters describing the probability of egg fertilisation, larval settlement, and post-settlement survival for the first year were sourced from the literature (see Table S1).

### Spatially explicit habitat parameterisation

A deterministic IPM assumes that vital rates remain constant over time, which we know is not the case, particularly over extended temporal periods^40^. Therefore, we required the capacity to modulate the intrinsic growth rate of the IPM in response to changes in the state of a population and the conditions at a site. Given that corals are sessile organisms, space is a limiting resource known to affect population growth rate. To simulate resource limitation through competition for space, the IPM was multiplied by the density dependent parameter, ${D}_{i,t}$ (Eq. 4), which accounts for the maximum coral habitat available at a site. Coral habitat is defined as the maximum percentage cover of corals that could occur across a given site (a comparable approach to that taken by Boschetti et al.^15^). In this way the coral habitat acts as a proxy for many interacting factors that influence the habitat suitability for corals.

The maximum coral habitat, as a percentage of the spatial area of each site, is calculated using satellite-derived benthic habitat maps^50^ (Supplementary Information 1.2). Each 10 m pixel in the benthic map is classified as one of four categories: Sand, Rubble, Rock or Coral/Algae (see Supplementary Information 1.2). According to Roelfsema et al.^50^, pixels defined as dominated by Coral/Algae and Rock can be considered as potential coral habitat with Coral/Algae being “any hardbottom area supporting living coral and/or algae” and Rock being “any exposed area of hard bare substrate”. As such, pixels classified to be dominant in either Coral/Algae or Rock were counted as suitable coral habitat. However, pixels are 10 × 10 m in size, and, due to the patchy nature of reefs at very fine scales, it is unlikely that the full area of the pixel is completely covered by the dominant classification. Hence, we utilised a replicate sampling strategy to account for the uncertainty of within pixel variation. For one of the four possible categories to be dominant, the dominant category would need to cover 26–100% of the pixel, while the non-dominant categories were $\le$ 25%. Therefore, we replicated the calculation of coral habitat 100 times, each time assigning a random value between 26 and 100% for the dominant category and 0–25% for the non-dominant categories using a uniform distribution. The mean coral habitat from this sampling was calculated as the proportion of potential coral habitat as a percentage. A three-dimensional coral area was determined for each site to consider sloping bathymetry which creates larger substrate area for corals relative to the two-dimensional area (Supplementary Information 1.3).

### Connectivity

#### Within-cluster connectivity

The workflow creates a spatial mosaic of site polygons, and each site represents a distinct area where a population of each of the two coral types is simulated. To establish a metacommunity comprising these populations, it was necessary to estimate the probability of coral larvae dispersing from one site to another. These probabilities are influenced by factors such as physical connectivity, including ocean currents and hydrodynamics during coral spawning^25^, as well as trait and behavioural characteristics of the coral larvae. Such probabilities could be obtained with various modelling or assumptions, but in this case, the eReefs RECOM model^80,81^ nested within GBR1^82^ was used to simulate transport and dispersal of larvae with a ~ 250 m spatial resolution^25^.

RECOM is a tool to nest local-scale models within regional-scale hydrodynamic-biogeochemical models of the GBR developed for the eReefs project^81^. RECOM produces a package of initialisation and boundary forcing files extracted from the eReefs environmental models, along with hydrodynamic transport files that can be used to drive more customised model runs. Coral larvae dispersal was simulated using passive tracers released at times that reflected the timing of annual mass spawning events for *Acropora* and *Goniastrea* spp., which typically spawn at the same time of year. Tracers were released on days 4, 5 and 6 following the full moon associated with known spawning events in 2015, 2016 and 2017. Releases were set to occur between 7:30 and 11 pm on each night of spawning from the centroid of each site polygon. Tracers were released at the water surface and were assumed to be neutrally buoyant. The ‘competency period’ of larvae was set to be between 4 and 28 days from release for both the corymbose *Acropora* and sub-massive coral^83,84^. The tracers were labelled according to their site of origin and tracked as they were transported by tides and currents through the local reef cluster region from release until the end of the competency period. A zero-gradient boundary was applied to tracer concentrations at the edge of the model domain (i.e., it was assumed that the concentration coming inwards across the boundary was the same as the concentration just inside the boundary).

We made various assumptions in a post-processing workflow to simulate coral larvae behaviour. We assumed larvae would settle in the first site they passed over during the competency period. At each hourly time step during this period, the total supply of each tracer was reduced by the proportion of tracer that was over a reef, and the cumulative quantity that had settled on each polygon was tracked. The total amount of tracers settling into each site was then converted into a probability matrix, one for each day of release and for each year simulated. Some tracers were lost from the reef cluster due to advection outside the modelled spatial domain. In the connectivity matrix, this was indicated by columns that did not sum to 1 and we assumed these larvae died or settled on reefs outside the cluster.

For the simulations explored in the present study we took the mean of the connectivity matrices for days and for years and used this mean matrix for all years.

#### External larvae supply

It may be reasonable to assume that a cluster of reefs being modelled is a closed system, but this depends on the spatial context. In our case study, the Great Barrier Reef is a well-connected system^5,6,85^ and there are generally other reefs surrounding potential reef clusters of interest, which likely share coral larvae with the reefs inside a chosen cluster. Therefore, for the simulations presented here, the ReefMod-GBR model^5^ was also run, for all reefs in the Great Barrier Reef, with the same environmental forcings (Supplementary Information 5). From the ReefMod-GBR model outputs we could then extract the number of larvae arriving to each reef in the Moore Reef Cluster in each of the years we simulated according to the ReefMod-GBR model outputs. These ‘external larvae’ were distributed to the sites proportionally based on the total site area, i.e., larger polygons received more larvae. In the same way as for larvae arising from within the cluster, these external larvae settled on the reef with a probability dependent on their coral type (Table S1), and then were governed by the IPM and the conditions at the site they were assigned to.

### Acute disturbances

Temperature stress, cyclones, and Acanthaster crown-of-thorns (COTS) starfish outbreaks are included as acute disturbances in the *C*~*scape* framework. In any given year these disturbances can impact the coral population by killing coral colonies within a site.

#### Temperature stress

Marine heatwaves cause temperature stress, coral bleaching and subsequent mortality^86^. Bleaching mortality can be predicted from Degree Heating Weeks (DHW), a metric of the accumulated heat stress^87^. We created a site-level DHW hindcast for our case study by combining information from two sources. We first sourced a reef-level DHW hindcast for each of the 5 reefs in our case study cluster by extracting the annual maximum DHW available at a 5 km resolution from the NOAA Coral Reef Watch (CRW) Product Suite version 3.1, as in^5^, from 2008 to 2022. Next, we modelled spatial variability in heat stress across the reef cluster in historic marine heatwave years using the eReefs RECOM model at a fine spatial resolution (250 m resolution). We did this for the period from 1 November in the preceding year through to 30 April in the named years for 2016, 2017 and 2020 (the period of most heat stress, Supplementary Information 3.1). We modelled surface DHW, as depth was handled separately in the coral mortality functions. From this modelling we assigned a DHW value to each site in the cluster for the three model years. We then calculated the mean DHW for each modelled heatwave year and each reef. From this we calculated the proportional residuals between the mean DHW of each reef and the DHW at each site (Supplementary Information 3.1, Fig. S10). We calculated the mean and standard error of the proportional residuals across the three modelled heatwave years and used these values as a scaling factor to apply to the full NOAA hindcast time-series for each reef (see Supplementary Information 3.1). Using the scalar in this way meant that the reef-level DHW hindcast was downscaled such that in any year taking the site average for a reef would give the reef-level DHW while creating site-level variability in temperature stress as determined from the finer-spatial scale RECOM modelling.

The probability of coral mortality in any given year was estimated as a function of DHW following the relationships developed in Bozec et al.^5^ based on observations of coral mortality during the 2016 bleaching event on the Great Barrier Reef^88^. Mortality is a function of the coral type, the DHW at a site and the depth of a site (see detail Supplementary Information 3.2).

The site polygons were overlaid on a bathymetric map of pixel size 10 × 10 m (https://www.eomap.com/) to determine the mean depth within each polygon, and all corals within each site were assumed to be at this mean depth (Fig. S2), which influences the temperature stress they experience (Supplementary Information 3.1).

#### Cyclones

*C*~*scape* is set up to receive a storm intensity for each reef defined on the Saffir-Simpson scale (1–5;^89^). Reef level exposure to cyclones can be difficult to determine as it depends on many factors including the distance of a reef from the cyclone path, the speed of the cyclone and fetch distances across which damaging waves can be generated^90^. For this study we assigned a cyclone category based on the observations in the AIMS LTMP which recorded cyclone damage at Moore and Thetford reefs in the year 2011 only. This was caused by Cyclone Yasi, and all reefs in the cluster were assigned a category 2 cyclone in this year, representing the category of Yasi when it passed the modelled cluster^91^.

To model coral mortality due to cyclones we fitted two regressions to the data in Fig. 6 of Fabricius et al.^92^, one for branching corals, and one for small sub-massive corals, to obtain coral mortality as a function of wind speed in m/s (Fig. S11). Windspeed was determined from cyclone category according to the Australian Bureau of Meteorology (Supplementary Information 3.2). We did not model variation in exposure to cyclones at the within-reef scale in the present study, nor consider how corals of different sizes may be influenced, but flag these as important areas for future model development.

#### Crown-of-thorns

COTS population dynamics are not modelled explicitly in *C*~*scape*, so we took outputs from the ReefMod-GBR model^5^ on the predicted COTS density per age class for each reef in the modelled cluster in each of the hindcast years. There were eight age classes of COTS with the abundance in each class given as density per m^2^. These values were multiplied by the total site area, such that larger sites received more COTS.

The function for coral mortality caused by COTS follows Bozec et al.^5^ (see Supplementary Information 3.3). We model coral mortality as a function of the abundance of COTS and age-dependent consumption rates. We incorporated a difference in the preference of COTS for the two different coral types, again following Bozec et al.^5^ based on information in De’ath and Moran^93^ which suggests corymbose *Acropora* is preferred over small sub-massive *Goniastrea* with a ratio of 14:4.3. Corals of all sizes experienced the same mortality, i.e., a proportion of the total coral colonies was removed by the COTS.

### Case study hindcast

To demonstrate the workflow for applying the *C*~*scape* framework to a reef cluster we chose a cluster of five reefs—Moore, Thetford, Milln, Briggs and Elford—on the Great Barrier Reef, offshore from Cairns (− 16.867°, 146.233°, Fig. 2).

#### Validation data

To determine whether the model framework could capture observed differences in total coral cover trajectories among sites, model predictions were compared to observed time-series of coral cover from the AIMS long-term monitoring program (LTMP^52,94^) available at two of the five reefs in the Moore Reef Cluster case study: Moore and Thetford reefs. Two datasets were available for these reefs: time-series of total coral cover from fixed-position photo transect data at three sites at on the leeward north-east slope of the reefs, and total coral cover estimates from manta tow surveys around the reef perimeters. Manta tow is a monitoring technique where an observer (on snorkel) is towed behind a boat and makes visual assessments, recording the average total coral cover in ~ 10 m wide bands on consecutive tows which are ~ 200 m in length^94^. The surveys follow the reef perimeter, usually on the reef slope^94^. The manta tow surveys are less accurate than the fixed-position photo transects but provide broader spatial information across the reef. Although designed to give reef level assessment, the manta tow data can be divided spatially into four reef sectors—back reef, flank 1, flank 2 and front (Fig. 2c, Miller et al.^94^)—which was useful for evaluating the within-reef variability simulated by *C*~*scape.*

#### Model simulations

For this case study we included inputs from the regional-scale model ReefMod-GBR^5^ to obtain predictions of external coral larvae and COTS densities in the modelled reef cluster (Table S4). ReefMod-GBR outputs were available from 2008 onwards, so we therefore initialised *C*~*scape* in this year and examined temporal dynamics through to 2022.

**Fig. 7:**
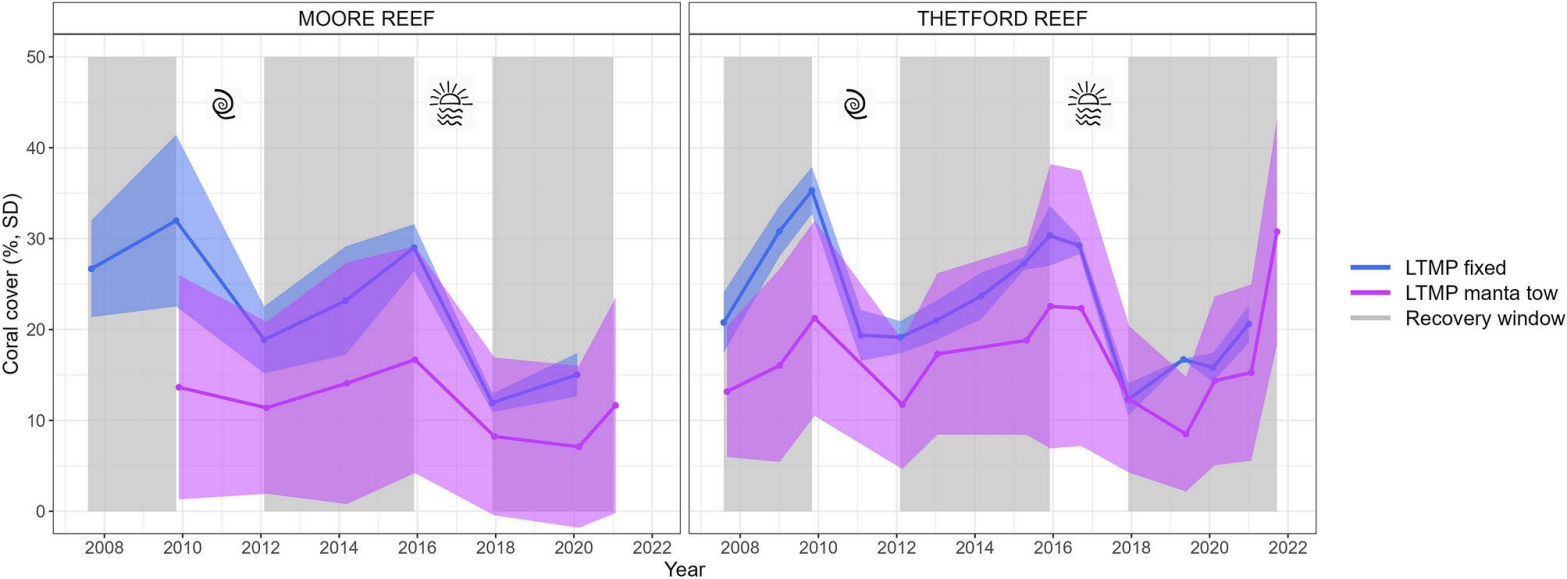
Temporal dynamics of the AIMS LTMP fixed-position photo transect and manta tow data in blue and purple, respectively. Mean and standard deviation are calculated from the three sites in the LTMP fixed-position photo transect data and across all available tows for the manta tow data. Two major disturbances occurred in the time-series, a cyclone in 2011 and a marine heatwave in 2017, as shown by symbols. Grey shading shows the ‘recovery windows’, i.e., time-periods identified to investigate population growth rate in the absence of acute disturbance.

Simulations were initialised based on coral cover recorded in the AIMS LTMP observations from the starting year of the hindcast: 2008 for the full trajectory, or the start of each ‘recovery window’ (Fig. 7) for the population growth rate analysis (see Supplementary Information 2.1).

We ran the model for two parameterisations of maximum coral habitat for each site in the reef cluster: uniform and site-specific coral habitat. For the uniform parameterisation of coral habitat, we tested a scenario where the maximum coral habitat was set at 80% for all sites. Various assumptions have been made regarding this upper limit in previous modelling studies (e.g. 100%^4,45^, 50–90%^5^, or it is sometimes informed from empirical field data^15,51^). For the second scenario, we developed a methodology for calculating a site-specific coral habitat parameter from satellite derived geomorphic and benthic maps. In this way we could investigate whether a site-specific parameter describing the availability of coral habitat could act as a useful proxy for the many processes that ultimately determine coral distribution and population growth. We wanted to contrast how using the available benthic habitats may improve upon a basic assumption of the maximum coral habitat.

We examined the full trajectory of the modelled hindcasts (for uniform coral habitat and for site-specific coral habitat calculated from the benthic maps) for all sites in each of Thetford and Moore reefs. We compared these trajectories to those observed in the manta tow and fixed-position photo transect time-series.

The manta tows were classified as one of four sectors. Therefore, we classified model sites as one of these four sectors: any polygons spatially underlying the manta tow tracks based on GPS tracks of the manta tow were assigned to the underlying manta tow sector, provided they were also classified as the reef slope or sheltered reef slope geomorphic zone, as the manta tows do not survey the outer flat or the crest zones (Fig. 2b,c). We visually compared trajectories of total coral cover in the empirical manta tow data for each sector with the predictions of total coral cover for the underlying *C*~*scape* polygons.

All scenarios were replicated 100 times, each sampling once from 100 the integral projection matrices generated to capture demographic uncertainty.

#### Time-averaged annual change in coral cover

Validating time-series dynamics can be challenging because, for instance, if a modelled disturbance early in the time-series leads to a perturbation that is either smaller or larger than observed in the empirical data, it introduces errors that propagate throughout the analysis. Therefore, we identified temporal windows without acute disturbances and calculated the time-averaged population growth rate as percent change in coral cover per year in these windows. We stipulated that these windows must span at least 2 years in duration. We identified three windows for each of the two reefs (Fig. 7). For each recovery window, we reinitialised the model using the coral cover data from the nearest empirical data point. This ensured that both the model and empirical growth rates were calculated from the same starting point.

We compared population growth rates as change in percentage coral cover over time for Moore and Thetford reefs for the: (i) Manta tow reef average versus the average of model sites underlying the tows spatially, (ii) Manta tow average in each of the four sectors versus the average of model sites underlying these sectors spatially (iii) Fixed photo transect average with the average of the three underlying or closest polygons (Fig. 2).

The manta tow GPS tracks crossed areas that the geomorphic maps indicated were not the appropriate habitat for corals (i.e., not one of Reef Slope, Reef Crest, Outer Reef Flat or Sheltered Reef Slope) and were therefore not modelled explicitly by *C*~*scape* in the case study. While we had GPS tracks of the manta tows, we did not have this information for each individual tow, and therefore could not remove the tows that were not over modelled reef areas for comparison to the model. Rather, we determined approximately how many site polygons would underlie the manta tow track if the geomorphic map had suggested it was coral-appropriate area. For Moore Reef this was eight sites in the back sector and for Thetford reef this was four sites in the back sector. For scenarios where we informed the model using the benthic habitat map, we specified that these sites have zero coral cover in all years.

## Supplementary Information

Supplementary Information.

## Data Availability

The model code and data used to run the *C~scape* model for the present study are accessible at https://apps.aims.gov.au/metadata/view/191db360-2009-49b6-b44f-606c228157f4.
