# Supplementary material for: Capturing fine-scale coral dynamics with a metacommunity modelling framework
Source: Sci Rep. 2024 Oct 21;14:24733. doi: 10.1038/s41598-024-73464-y (PMC11494194; doi:10.1038/s41598-024-73464-y)
Supplement: Supplementary file 1 — Supplementary Information. [file 41598_2024_73464_MOESM1_ESM.pdf]

Supplementary information for

Capturing fine-scale coral dynamics with a metacommunity modelling framework

Anna K Cresswell[1,2,*], Vanessa Haller-Bull[3], Manuel Gonzalez-Rivero[3], James P Gilmour[1,2], Yves-Marie Bozec[4], Diego R Barneche[1,2], Barbara Robson[3,8], Kenneth R N Anthony[3], Christopher Doropoulos[5], Chris Roelfsema[4], Mitchell Lyons[6], Peter J Mumby[4], Scott Condie[7], Veronique Lago[3,8], Juan-Carlos Ortiz[3]

[1] Australian Institute of Marine Science, Perth, WA, Australia, 6009

[2] Oceans Institute, University of Western Australia, Perth, WA, Australia, 6009

[3] Australian Institute of Marine Science, Townsville, QLD, Australia, 4810

[4] School of The Environment, The University of Queensland, Brisbane, QLD, Australia, 4072

[5] CSIRO Environment, St. Lucia, QLD, Australia, 4067

[6] University of New South Wales, Sydney, NSW, Australia, 2052

[7] CSIRO Environment, Hobart, Tasmania, Australia, 7001

[8] AIMS@JCU (aims@jcu.edu.au), Townsville, Queensland, Australia, 4810

* Corresponding author: annacresswell@gmail.com, ORCID: 0000-0001-6740-9052

Contents

Supplementary Information 1: Generating a spatially explicit seascape
Supplementary Information 2: Integral Projection Models
Supplementary Information 3: Acute disturbances
Supplementary Information 4: Moore Reef Cluster case study
Supplementary Information 5: Summary of inputs used to run C~scape simulations

1. Generating a spatially explicit seascape

1.1. Delineating sites

As discussed in the main text, it was necessary to divide each reef(s) into units. We used a geomorphic map from Roelfsema *et al.* (2020) sourced from the GBRMPA Reep Authority Geohub https://geohub-gbrmpa.hub.arcgis.com/. The geomorphic map was created using a combination of machine learning and semi-automated expert-driven contextual editing (Lyons *et al.* 2020). A random forest classifier used expert-curated training data to predict geomorphic zones from a stack of Sentinel-2 satellite imagery (10 m pixels) and other physical attributes (depth, slope, wave environment parameters), the output of which was then contextually edited via an expert-driven ruleset.

Following the recommendations of Kennedy *et al.* (2021), we only considered the geomorphic zones that are expected to have predominantly hard substrate — Reef Slope, Reef Crest, Outer Reef Flat and Sheltered Reef Slope — to be areas where we would expect appropriate habitat and conditions for corals to grow (description in table S1, Roelfsema *et al.* 2021). While other zones may have patchy coral habitat, we considered this to be minor in terms of potential to contribute to the metapopulation, as it is likely that distinct species of corals occur in these more marginal habitats.

Figure S 1 illustrates the process of pixelating each geomorphic zone as detailed in the main text. Figure S 2 shows the Moore Reef Cluster partitioned into sites for modelling.

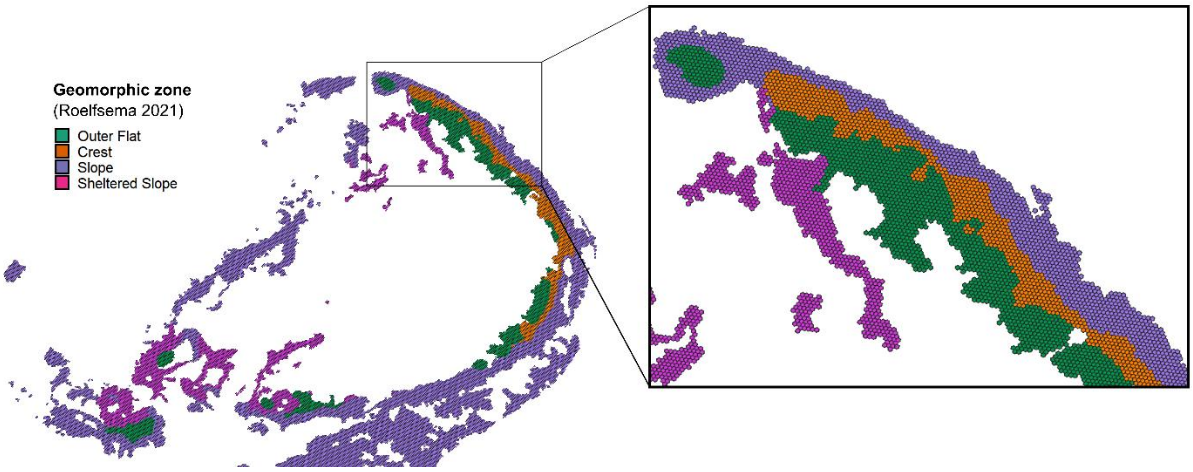

Figure S 1. Moore Reef with the four selected geomorphic zones — Reef Slope, Reef Crest, Outer Reef Flat and Sheltered Reef Slope — pixelated into hexagons in preparation for clustering.

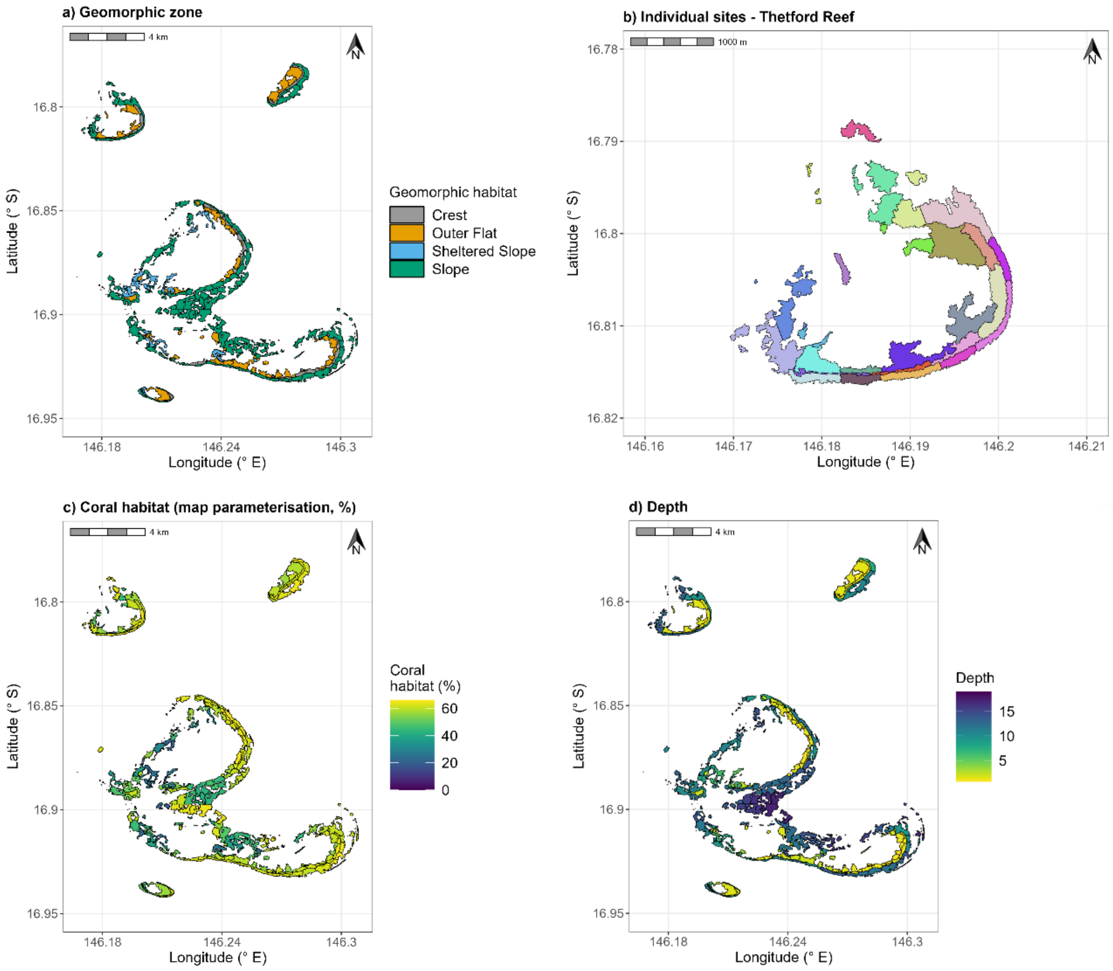

Figure S 2. a) The Moore Reef Cluster partitioned into site polygons, coloured by geomorphic zone. b) Sites in Thetford Reef (north-west in Moore Reef Cluster) showing 27 individual sites. Each site is modelled discretely in C~scape and connected to the other polygons via the transport of coral larvae. Note that some polygons are 'multi-polygons', i.e., made up of >1 polygon, but still classified as one site for the modelling units. c) Sites coloured by their maximum coral habitat when parameterised by habitat maps. d) Mean depth (metres) of each polygon.

Reef	No. sites
Briggs	7
Elford	95
Milln	15
Moore	69
Thetford	27
**Total**	**213**

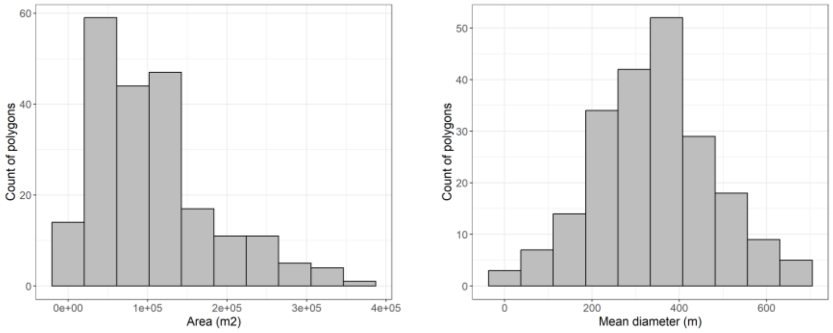

Figure S 3. Summary of the number of site polygons in each reef and histograms showing the size of the 213 modelled sites in total area and diameter (when polygons are assumed to be circular). The mean diameter was 340 m2.

1.2. Parameterising site-specific coral habitat

The benthic habitat map from Roelfsema *et al.* (2021) were used to calculate the proportion of coral habitat in each site. The benthic habitat map was created using the same underlying framework and input data sets as the geomorphic zonation map (Roelfsema *et al.* 2021). The key difference is the machine learning model was trained using point-based field observations and the contextual editing process was able to use the geomorphic map as an input layer.

Each site polygon was assigned a coral habitat value to represent the percentage of the total polygon area where coral could potentially grow. The benthic habitat maps are composed of 10x10m pixels classified as one of four categories: Sand, Rubble, Rock, Coral/Algae (main text, Figure S 4).

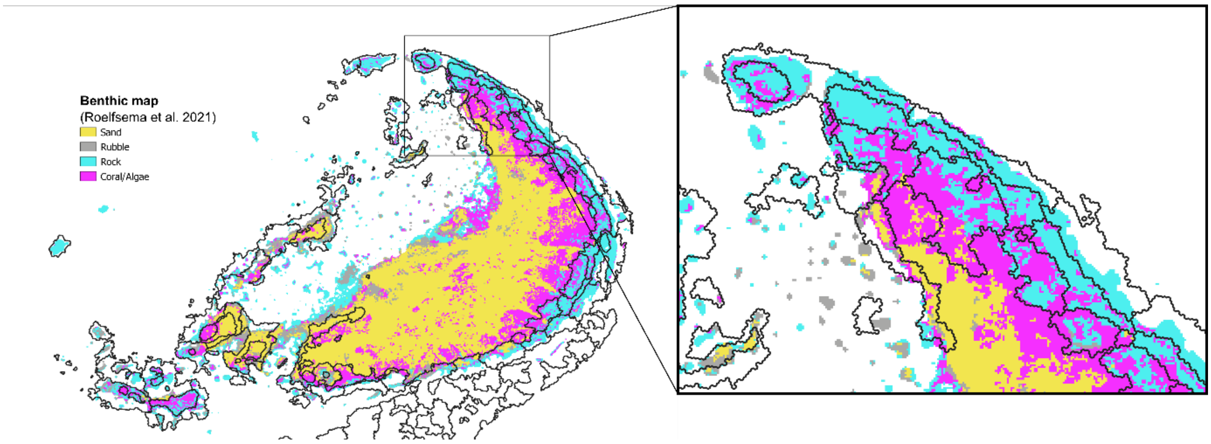

Figure S 4. The benthic reef habitat map from Roelfsema et al. 2021 showed by four distinctly coloured benthic classifications for Moore Reef, overlaid with the site polygons delineated from the geomorphic map in black. Inset shows a section in the north east corner of Moore Reef.

The benthic habitat map did not always cover the full extent of the geomorphic habitat map, meaning that there were pixels within the site polygons that had no benthic map information, i.e., NA values. These NA values were removed from the calculation of coral habitat. In cases where more than 95% of the pixels in any given polygon were NA, we instead took the value of coral habitat from the nearest neighbour polygon that had the same geomorphic classification.

All site polygons have a spatial area defined in square metres, $Area_i$, which varies depending on their size and shape. Importantly, this is distinct from the ‘potential coral area’ that is modelled, which can be calculated as the sum of each sites area multiplied by its maximum coral habitat, K_i, as a proportion of its total area:

$$Area_{reef} = \sum_{i=1}^{n\ sites} Area_i \times K_i$$

1.3. 3D reef area

Using the geomorphic maps to create the site polygons determines the total reef area which is modelled across the seascape. Assigning a coral habitat value to each site then sets an upper limit on the amount of coral area across the reefs. We converted the 2D site polygon areas from the map to 3D surface area, using a “surface-to-horizontal-area ratio”, derived from the slope value estimated for each pixel. Slope was estimated by using a local gradient method (3 × 3 window) from a bathymetric map. The slope-adjusted surface area, i.e., 3D surface area for each pixel, was calculated using a trigonometric formula of 2D surface area and the slope where

$A_{3D} = A_{2D}/\cos(slope)$, where the slope is in radians.

The 3D area for each site was the sum of all pixels 3D area within the site. The total 3D area of site polygons for Moore Reef Cluster was 22588866 m^2 = 2259 hectares, but not all of this was available for coral, as governed by the coral habitat. If coral was at maximum coral habitat in all site polygons in the Moore Reef Cluster this equated to 1207 hectares of coral.

1.4. Connectivity

The eReefs RECOM model (Herzfeld 2009; Steven *et al.* 2019) nested within GBR1 (Skerratt *et al.* 2023) was used to simulate transport and dispersal of larvae with a ~250 m spatial resolution. This allowed for the generation of a connectivity matrix which captured variability in the number of larvae retained in a site, exported to other sites, and received from other sites (Figure S 5). Some reefs (e.g., Thetford Reef, northwest) were found to be strongly self-seeding, while other reefs (e.g., Elford and Moore Reef) shared larvae with each other (Figure S 5).

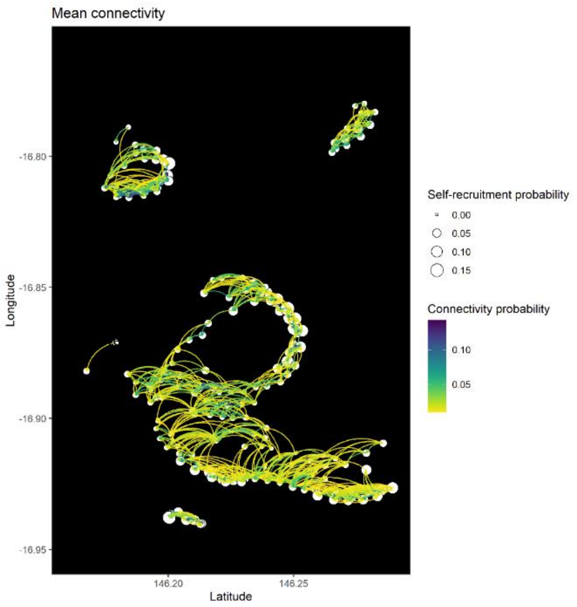

Figure S 5. Visualisation of connectivity. Sites are represented by white points with the size of the points indicating the proportion of larvae remaining within the source site polygon. The colour of lines indicates the proportion of larvae travelling between site polygons, while

2. Integral Projection Models

2.1. Mathematical construction

An Integral Projection Model (IPM) is a continuous-state, discrete-time model (Easterling et al. 2000) and is a form of an integrodifference equation (Neubert et al. 1995), where the spatial re-distribution is replaced by a size re-distribution. The IPM consists of two components: the state variable and a transition matrix.

The state variable of the IPM describes the number of individuals $n(x,\ t)$ at year t with state x. In this work, x and y represent the size as colony area of individual coral colonies as well as three additional discrete states: egg, larvae and settler such that:

$$n(x,\ t) = [n_{egg,t} \quad n_{larvae,t} \quad n_{settler,t} \quad n_{x\,1,t} \quad n_{x\,2,t} \quad n_{x\,3,t} \cdots \quad n_{x\,n-1,t} \quad n_{x\,n,t}]$$

This describes the abundance of coral eggs, larvae and settlers (the discrete states) and the size structure of the coral population at one point in time. The number of size classes, n, is user defined (see discussion of discretisation below).

The transition matrix, or kernal $k(y,x)$, is analogous to a projection matrix (e.g. Leslie matrix, (Hansen 1989)) in matrix population modelling (Caswell 2000). It represents all possible transitions from state x (in year t) to state y (in year $t+1$), integrated over all states of x (Metcalf *et al.* 2013). The mathematical definition of the kernel is flexible but was here defined as the product of survival of individuals in state x at time t, $s(x)$, and growth of individuals from state x to state y, $g(x,y)$, plus the fecundity of individuals in state x producing those in state y, $f(x,y)$, at time t+1.

$$k(y,x) = s(x)g(x,y) + f(x,y)$$

Survival, $s(x)$, is the probability that an individual of size x at time t will be alive at time t+1. Growth, $g(y,\mathrm{x})$, is the probability that an individual of size x at time t will grow to an individual of size y at time t+1. This function considers cases of positive growth where $y > x$ and also negative growth, i.e., partial mortality of corals, where $y <$ x. The coral species we included generally did not fragment into multiple individual ramets, hence this was not captured in the demographic data and was not included in the models.

Similar to the growth and survival functions, fecundity, $f(x,y)$, is determined as a function of coral colony size. We assume that once a coral individual is large enough to reproduce, it produces eggs once each year (once per model time step) until the individual dies.

The number of eggs that are produced by a coral colony is modelled as

$$f(x) = A\,\rho\,E\,m$$

Where A is the colony surface area in cm^2, ρ is the density of coral polyps (cm^{-2}) (Doropolous et al. 2020), E is the number of eggs per polyp (Pratchett et al. 2019; Doropolous et al. 2020), and m is the proportion of these polyps that are mature (Alvarez-Noriega et al. 2016, Doropoulos et al 2020).

The kernel $k(y,x)$ can be used to predict the number of individuals $n(y,\ t+1)$ at year $t+1$ with a given state y as a function of the number of individuals $n(x,\ t)$ at year t with state x.

$$n(y,\ t+1) = \int_L^U k(y,x)n(x,t)dx$$

Discretisation is necessary to facilitate the numerical evaluation of the integral. A meshpoint, x, serves as the mid-point for each bin in the discretisation, representing a size class within the lower (L) and upper (U) size of the coral. We used 1 cm diameter for the lower limit for the continuous state (capturing corals smaller than this in the 'settler' discrete state). The upper diameter is set at 90% of the maximum size observed in the data at the beginning of the sampling period to avoid statistical predictions at the limits with few samples. There is a trade-off between using a high number of meshpoints (m), which should give greater accuracy, and the computational cost of a high number of meshpoints. In the present study we used 100 meshpoints, which were evenly distributed on the log-scale of coral colony surface area.

The width of a given meshpoint can be calculated as $\Delta x = \frac{(U-L)}{m}$. Combining this with the midpoint rule (Ellner & Rees, 2006) the numerical evaluation results in

$$n(y, t+1) = \Delta x \sum_{i=1}^{m} k(\mathrm{y}, \mathrm{x},) n(\mathrm{x}, t)$$

Which gives a matrix, *m* by *m,* here 100 by 100, that represents the continuous state transition matrix (Metcalf *et al.* 2013).

The discrete transitions must also be parameterised, i.e., the transition probability from eggs to larvae, larvae to settlers, and settlers into the continuous state (see Figure S 6).

2.2. The coral life cycle

An IPM requires parameterisation of transitions between the main stages in an organism's life cycle over a temporal period, here annual, such that changes in the abundance and size, and mortality and recruitment to a population can be synthesised into a transition probability matrix. Figure S 6 gives a schematic summary of the coral life cycle as modelled in this study.

Growth, survival, and fecundity were modelled as a function of coral size as a continuous variable, but we also required several discrete transitions. Therefore, we included discrete states for coral egg, larvae, and settler to close the life cycle and complete the IPM (Merow *et al.* 2014). As in many demographic studies (Elderd & Miller 2016), early life history is relatively poorly understood for corals, despite the importance of processes such as fertilisation, settlement, and survival of recruits in determining population success (Hughes & Jackson 1985; Babcock 1991; Doropoulos *et al.* 2015). We parameterised these transitions with information from the literature (see Table S 1).

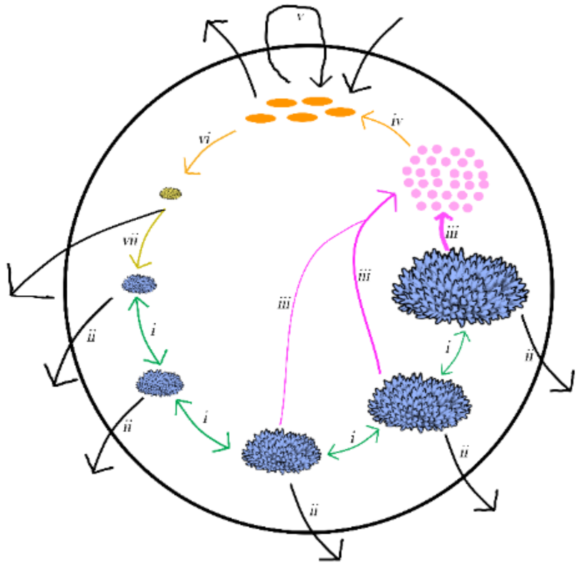

Figure S 6. A schematic representation of the coral life cycle and the main modelled processes. Different sized coral colonies are represented in the lower hemisphere of the circular life cycle, as well as an egg, larvae and settler state in the upper hemisphere. In any given timestep a coral may die (ii, survival), or else may transition to a different size (i, growth), either growing, shrinking (partial mortality) or staying the same size, with probabilities determined by statistical Bayesian regressions of growth and survival. Mature colonies may reproduce once in a timestep (iii), releasing gametes into the water column which may fertilise to become larvae (iv). Transport of these larvae is then simulated separately via connectivity modelling, before these larvae may settle within a site to become a settler (vi), before transitioning into the continuous state one year later (vii). The approach for modelling these processes and the data used are detailed in Table S1.

2.3. Vital rate regressions: growth and survival

To construct the integral projection matrices, growth, survival and fecundity functions are obtained from statistical regressions built using empirical data from monitoring coral colonies over annual timesteps (growth and survival functions) and surveying the number of eggs and polyps in coral colonies (fecundity function).

We fit the models for each of growth, survival, and fecundity as a function of coral size in R using the brms package (Bürkner 2017). We used multilevel models to predict the three continuous vital rates as a function of coral colony area (cm^2). We followed the approach of Elderd and Miller (2016) and Kayal *et al.* (2018) to fit the models using a Bayesian framework.

Colony growth was modelled by predicting coral colony surface area at time t+1 as a function of coral colony surface area at time t, on a natural log-log scale, assuming a Gaussian distribution with identity link. We included a random effect for colony ID, to account for some colonies being measured over multiple years. We included a fixed effect for dataset for the corymbose *Acropora* model. *Goniastrea* data was only available from Scott Reef and so we did not require a fixed effect for this variable.

Survival models were fitted using a Bernoulli distribution (and a logit link) with binary output of alive or dead at time t+1 as a function of colony area (on the natural log scale) at time t. Fixed and random effects were incorporated as for the growth models.

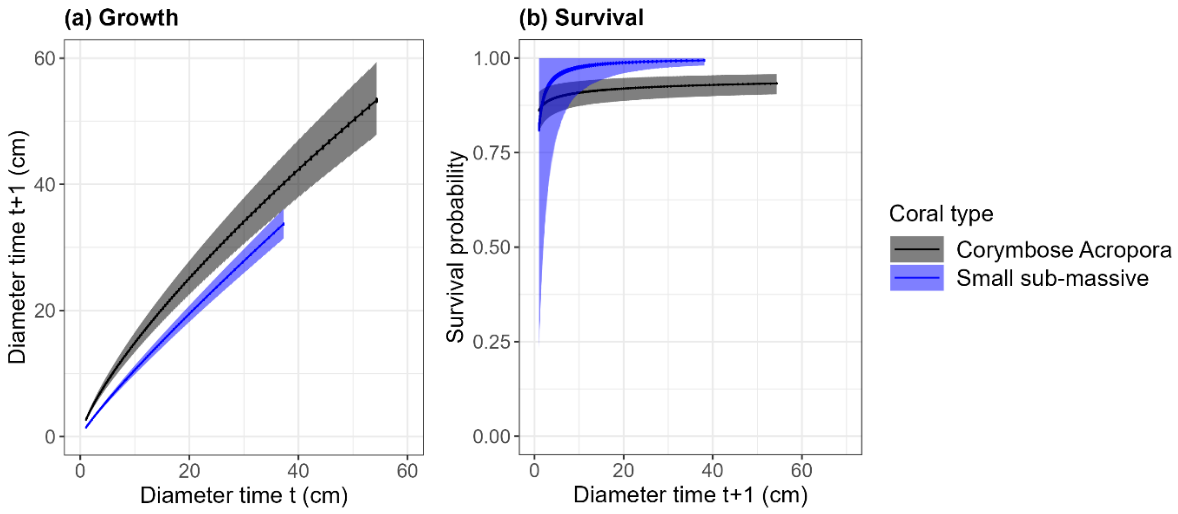

Figure S 7. Predictions from growth and survival statistical regressions. a) Predicted diameter at time t+1 as a function of diameter at time t and b) predicted probability of surviving the annual period as a function of coral colony diameter at time t. Predictions are shown in grey for corymbose Acropora and blue for the small-massive Goniastrea coral types. Solid line shows the mean and the ribbon shows the 95% Credible Interval.

2.4. Vital rate regressions: fecundity

Fecundity (the number of eggs, E, produced by a coral colony) was modelled as a function of colony area. We used a Hurdle-Poisson model (with a log link) which could account for small colonies being non-reproductive. Hurdle models are commonly used to model zero-inflated ecological data (Balderama et al. 2016; Brown et al. 2016; Cunningham et al. 2018) and consist of two components. The first component is the zero-inflated model, which describes the probability of coral being reproductive or not. The second component is the conditional model, which uses a zero-truncated error distribution to describe the relationship between model predictors and non-zero egg count data.

We separately fitted a model the number of polyps per cm^2 colony surface area, ρ as function of coral size in cm^2 (log-transformed) with a gamma distribution and a log-link, to account for larger colonies potentially having more or less polyps per surface area.

Multiplying predictions from these models was used to estimate the number of eggs as a function of colony size (Figure S 8).

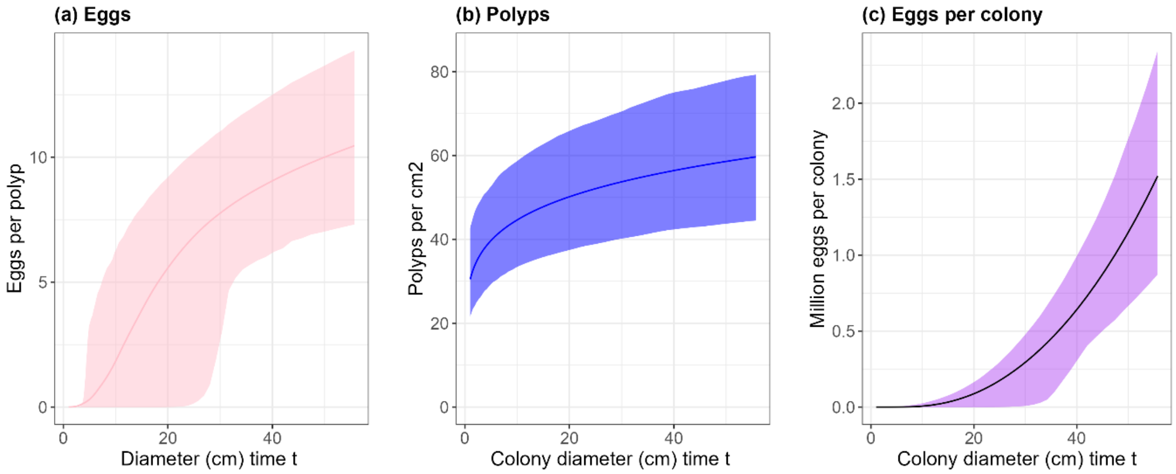

Figure S 8. a) Eggs per polyp as a function of coral colony size. Notably, at approximately 10 cm diameter colonies start to become reproductive. b) polyps per cm2 of coral area as a function of colony size. c) multiplying the predictions from the regressions in a) and b) gives a prediction of the number of eggs produced per colony of a given size. Solid line shows the mean, and the ribbon shows the 95% Credible Interval. These results were used for both corymbose Acropora and for the small-massive Goniastrea coral types.

2.5. Capturing uncertainty from the Bayesian regressions

Predicting from the regressions allows the creation of integral projection matrices. We sampled growth, survival and fecundity predictions from the joint posterior distributions of the model 100 times to maintain a measure of uncertainty in vital, i.e.:

$$k(y,x)_j = s(x)_j g(x,y)_i + f(x,y)_j$$

where $k(y,x)_j$ is a kernel or integral projection matrix, $s(x)_j$ is survival of individuals in state x from time t to t+1, $g(x,y)_\mathrm{j}$ is the growth of individuals from state x to state y, $f(x,y)_\mathrm{j}$ is the fecundity of individuals in state x producing those in state y at t+1, and *i* is a posterior sample.

2.6. Discrete transitions

Probabilities for egg fertilisation to larvae, and larvae settlement probability were informed from the literature (see Table S 1).

The transition from settler to the continuous state of the IPM required data on what size the settler corals were likely to be 1-year following settlement, as this would define their size, $n(y,\ t+1)$. To parameterise this we used data from Cruz and Harrison (2017), Dela Cruz and Harrison (2020) and Harrison *et al.* (2021) who measured the survival and growth of corals that settled on tiles and natural substrates over ~3 years.

To parameterise the size at which corals would transition from settler into the continuous state of the integral projection matrix we generated their randomly from a normal distribution with mean value 2.56 cm for acroporids and 1.41 cm for non-acroporid species according to Cruz and Harrison (2017), Dela Cruz and Harrison (2020) and Harrison *et al.* (2021). We used a standard error of 0.1 times the mean diameter.

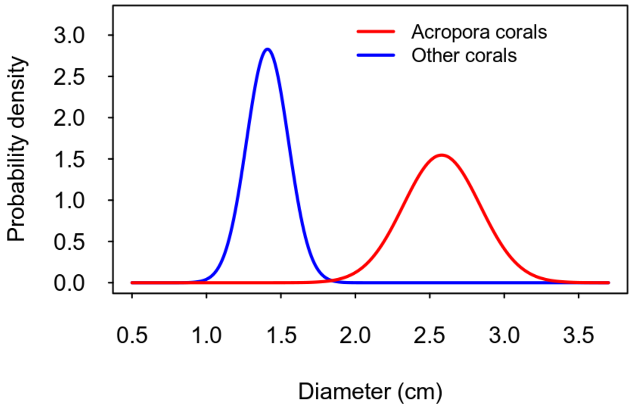

Figure S 9. Probability density plot showing the distribution which was sampled to determine the probability of transitioning to each size class in the integral projection matrix 1 year after settling.

Table S 1. Summary of the vital state transitions modelled in the Integral Projection Models to capture the life cycle shown in Figure S 6. The data and approach used to model or parameterise the transition is detailed and references are provided.

Vital rate transition	Process	Data used to	Parameters/ data corymbose *Acropora*	Parameters/ data small sub-massive *Goniastrea*	Reference(s)
i	Growth/ Shrinkage (partial mortality)	Fit regressions for $\mathrm{size}_{t+1} \sim \mathrm{size}_t$	Growth data encompassed a set of broadly comparable *Acropora* species across three datasets. Data from 2329 coral colonies from Scott Reef[a] (*A.millepora*), 2045 from Moorea[b] (*Acropora hyacinthus, A. globiceps*, *A. retusa*, and *A. fragilis*), 202 from Heron Island, GBR[c] (colonies <5cm diameter at time t: *A. hyacinthus, A. nasuta, A. humilis, and A.* spp). Site-year combinations that had known acute disturbances in the Scott Reef dataset were filtered out a priori.	Data for *Goniastrea* was sourced from the Scott Reef dataset only and included *G. retiformis* and *G. edwardsii*. Data was obtained from 1054 coral colonies[a]. Site-year combinations that had known acute disturbances were filtered out a priori.	[a] Gilmour et al. (2013) [b] Kayal *et al.* (2018) [c] Doropoulos et al. (2015)
ii	Survival	Regressions for $\mathrm{survivalstatus}_{t+1} \sim \mathrm{size}_t$	As above.	As above.	As above.
iii	Fecundity: eggs per polyp	Regressions for number of eggs per polyp as function of colony size $F_{oocyte_{t+1}} \sim size_t$	Data was taken from on average 5 polyps from 3 separate branches from 120 A. millepora coral colonies total from Scott Reef.	As for Acropora.	Gilmour et al. (2013) Foster and Gilmour (2020)
	Fecundity: polyps per cm² coral area	Fit regressions for number of polyps per colony surface area as function of colony size $F_{polyp_{t+1}} \sim size_t$	Data was taken from 120 A. millepora coral colonies total from Scott Reef.	As for Acropora.	Gilmour et al. (2013) Foster and Gilmour (2020)
	Fecundity: colony	$F_{colony_{t+1}} \sim F_{oocyte}(size_t) \times F_{polyp}(size_t)$	As above.	As above.	As above.
	Polyp sexual maturity	Parameterise the proportion of mature polyps per colony. Multiplied by $F_{colony_{t+1}}$ to account for unmature polyps within a colony.	40% of polyps assumed to contain fertile eggs.	As for Acropora.	Doropoulos *et al.* (2019) Álvarez-Noriega *et al.* (2016)
iv	Fertilisation to larvae	Parameterise fertilisation probability: multiplied by $F_{colony_{t+1}}$ to determine number of larvae.	55% of spawned eggs assumed to get fertilised in the water column.	As for Acropora.	Oliver and Babcock 1992 .
	Early mortality prior to dispersal	Parameterise larvae mortality: multiplied by $F_{colony_{t+1}}$ to determine number of larvae.	50% of larvae assumed to die in first three days during dispersal, before becoming competent to settle (e.g. predators, natural mortality)		Graham, E. M., Baird, A. H., & Connolly, S. R. (2008).
v	Larvae transport, export and import	Connectivity modelling conducted to create connectivity matrix describing probability of moving among sites.	See main text	See main text	
vi	Settlement	Parameterise the probability that a larvae will settle if it is over reef within its competency window.	From the lower and upper limits of larvae settlement probabilities[d,e] we took the mean probability of settlement for Acropora to be 2.1% ((0.05-0.008)/2).	The settlement probability of Goniastrea was assumed to be 60% that of Acropora at 1.26%[f]	[d] Edwards et al. 2015 [e] De La Cruz and [f] Harrison (2017), [g]Wallace (1985)

Table S 2.continued

Vital rate transition	Process	Data used to	Parameters/ data corymbose *Acropora*	Parameters/ data small sub-massive *Goniastrea*	Reference(s)
vii	Growth to 1-year old coral	Parameterise the probability of transitioning from the discrete ‘settler’ state to the continuous state.	1.315%	1.315%	Doropoulos *et al.* (2019)
vii	Size at 1-year old	Used to specify the size of corals 1 year after settlement. It is at this point that they transition into the continuous state of the IPM and must be assigned a size.	Sampled from a normal distribution with mean 2.56cm and standard deviation 0.26cm.	Sampled from a normal distribution with mean 1.41cm diameter and standard deviation 0.14cm	Cruz and Harrison (2017)<br>Dela Cruz and Harrison (2020)<br>Harrison *et al.* (2021)

3. Coral population mortality agents

3.1. Temperature stress

Temperature stress spatial variability

Site-level downscaling was conducted for heat stress. Variability in heat stress across the reef cluster was modelled in historic marine heatwave years. The eReefs RECOM model (Herzfeld 2009; Steven *et al.* 2019) nested within GBR1 (Skerratt *et al.* 2023) was used to implement a hydrodynamic model in SHOC (Herzfeld 2009) with a grid resolution of approximately 250 m within the 1 km resolution version 2.0 GBR-scale hydrodynamic model. SHOC can be used to calculate two- or three-dimensional DHWs given a reference climatology. For this project, we used the 4 a.m. DHW product using the STTAARS regional climatology (Wijffels *et al.* 2018). While this product is not entirely consistent with the NOAA Coral Reef Watch product, and an improved approach is needed in future work, it was used only to estimate the relative differences between grid-cells on the local scale, for local-scale adjustment of regional DHW projections.

RECOM was run for the period from 1 November in the preceding year through to 30 April in the named years for 2016, 2017 and 2020 (years with historically higher acute thermal stress). We modelled surface DHW, as depth was handled separately in the coral mortality functions (see section ‘temperature stress mortality in C~scape’). From this modelling we assigned a DHW value to each site in the cluster for the three modelled heatwave years. We then calculated the mean DHW for each modelled heatwave year and each reef. We calculated the proportional residuals (R) between the mean DHW value of a reef and all sites within the reef for the three marine heatwave years that were modelled using RECOM:

$$R_{i,y} = \frac{DHW_i - \frac{\sum DHW_i}{n}}{\frac{\sum DHW_i}{n}}$$

where i=1 to n for each of the 213 sites in the Moore Reef Cluster and y is each of the marine heatwave years (2016, 2017, 2020).

Next, the mean, $mean(R_i)$, and standard deviation, $std(R_i)$, of the residuals were calculated for each site across the three years (Figure S 10) to allow the fitting of a normal distribution. These values were then used as a scaling parameter to downscale the reef level hindcast timeseries from NOAA for which there was only a single value for the reef. Calculating a scaling parameter in this way meant that in any year taking the average of all the sites DHW values for a reef would give the reef-level DHW, while creating site-level variability in temperature stress, as determined from the finer-spatial scale RECOM modelling.

Using the scaling parameters we calculated $DHW_{i,t}$ for each site *i* and for each time *t* for which we had a hindcast value for the DHW experienced at the reef level ($DHW_{reef,t}$) from NOAA:

$$DHW_{i,t} = DHW_{reef,t} + DHW_{reef,t} * rnorm(mean(R_i), std(R_i))$$

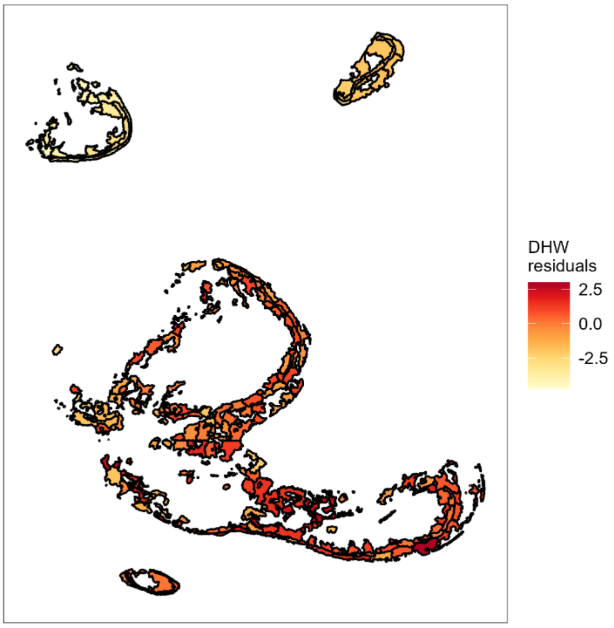

Figure S 10. The mean Degree Heating Week residuals across the three marine heatwave years examined illustrating how spatial variation in mean heat exposure is implemented in the C~scape framework.

Temperature stress mortality in C~scape

The probability of coral mortality in any given year was estimated as a function of DHW following the model developed in Bozec *et al.* (2022) based on observations of coral mortality during the 2016 bleaching event (Hughes *et al.* 2018). The data available from (Hughes *et al.* 2018) data could be divided into two types: (i) initial bleaching mortality recorded at the time of peak temperature stress and (ii) long-term mortality observed after 6 months. Due to the yearly time-step in C~scape, long-term mortality was the most appropriate metric to use, but was in the form of a change to coral cover. Initial bleaching data was at the colony level, which was more appropriate given the IPM engine of C~scape. Therefore, we used both data types following the approach of Bozec *et al.* (2022) and calculated bleaching mortality in two main steps.

First, mortality at the peak of a bleaching event was calculated using a model of initial mortality as a function of DHW, fitted to the data from figure 2a Hughes *et al.* (2018) and based on the equations developed for ReefMod-GBR (Bozec *et al.* 2022).

Two additional coefficients were added to the model of initial mortality such that

$$m_{init_{ft_{site}}} = w_{site} \cdot s_{ft} \cdot \left(\left(e^{0.17+0.35 \cdot DHW_{site}}\right) - 1\right)/100$$

with w being the depth coefficient and s being the bleaching sensitivity of coral type *ft*.

The bleaching sensitivity coefficient was included because the data from figure 2a, Hughes *et al.* (2018) was from multiple different coral types and we needed to apply it more specifically to the coral types modelled in the present study so we followed Bozec *et al.* (2022) approach here.

The depth coefficient was included because data in figure 2a, Hughes *et al.* (2018) was collected at roughly 2m depth and is not representative of all depths.

Another piece of information from Baird *et al.* (2018), showing the relationship of bleaching with depth (fig 2, (Baird et al. 2018), was included to obtain depth coefficient w. We fit an exponential model to the data from fig 2, Baird *et al.* (2018), to get a formula to calculate the depth coefficient w using the depth of each site.

$$w_{site} = e^{-0.07551(sitedepth-2)}$$

The sensitivity for each coral type was taken from table S1, Bozec *et al.* (2022): the scaling was 1.4 for corymbose / small branching Acroporids (here used for corymbose *Acropora*) and 0.25 for small sub-massive corals (here used for small sub-massive *Goniastrea*).

The initial mortality of each coral at each site is therefore calculated as

$$m_{init\,ft_{site}} = w_{site} \cdot s_{ft} \cdot \left((e^{0.17+0.35 \cdot DHW_{site}}) - 1 \right) / 100$$

$m_{init\,ft\_site}$ was capped at 1.

Annual mortality (M) was then calculated based on data from figure 2c Hughes *et al.* (2018) and work by Bozec *et al.* (2022) who calibrated the long-term bleaching mortality to the initial bleaching mortality by combining information from figure 2a and 2c in Hughes *et al.* (2018).

$$M_{ft\_site} = 1 - (1 - m_{init\,ft\_site})^6$$

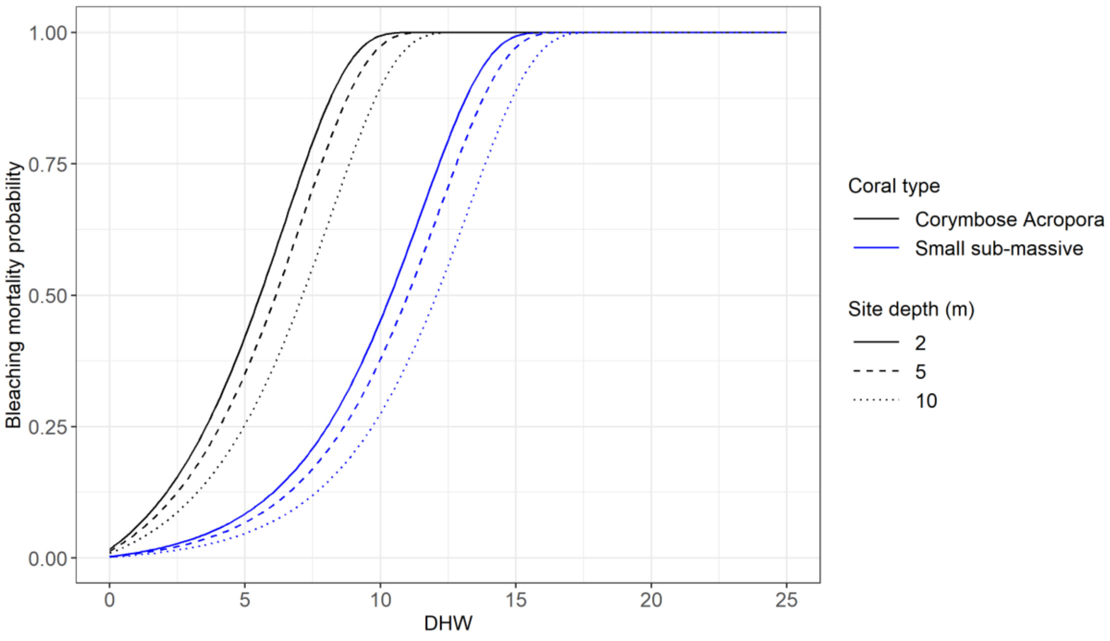

Mortality was imposed as a percentage reduction in coral cover, imposed equally across all coral sizes.

3.2. Cyclones as mortality agent

Each reef received a cyclone value for every year parameterised as an integer value from 0 to 5, but these were converted to windspeed so that information from Fabricius *et al.* (2008) could be used to estimate resulting mortality.

Table S 3. Cyclone categories and their associated windspeed, according to the Bureau of Meteorology, used to make a conversion from cyclone category to windspeed.

Cyclone category	Windspeed
1	24.5 m/s
2	32.5 m/s
3	44.2 m/s
4	55.3 m/s
5	65 m/s

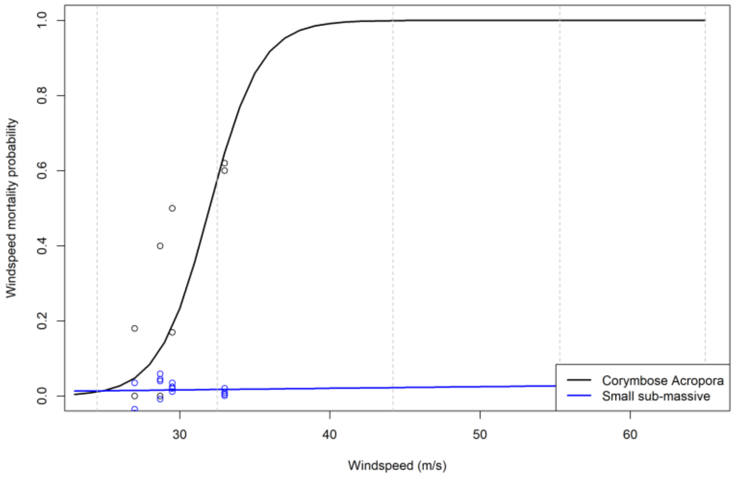

Figure S 11. Windspeed and the predicted associated probability of coral mortality for the two coral types included in this study: corymbose Acropora and small sub-massive Goniastrea. Dashed vertical lines indicate cyclone categories 1-5 from left to right.

Cyclone mortality was imposed as a percentage reduction in coral cover, imposed equally across all coral sizes.

3.3. Crown of thorns starfish as a mortality agent

We calculated the feeding on corals by COTS and the resulting coral mortality following the approach in Bozec *et al.* (2022). This approach was informed by published rates of consumption based on the size of COTS in Keesing and Lucas (1992), converted to the eight age classes using information in Engelhardt *et al.* (2001).

To include COTS consumption selectivity between coral types, information from De'ath and Moran (1998) is utilised. According to this data, *Acropora* is preferred over *Goniastrea* at an odd ratio of 14:4.3.

4. Moore Reef Cluster case study

4.1.Simulation details

Table S 4. Details of model simulations. All simulations in the table were repeated twice, once for each of the two parameterisations of coral habitat. Simulations were started in different years reflecting the start of each recovery window (Figure 7 main text) to obtain the time-averaged annual change in coral cover.

Dataset for validation and initialisation	Year initialised	Location of initialisation data	Recovery windows examined for reef and reef sector
Fixed-position photo-transects	2008 *	Moore & Thetford slope	2008-10 Moore<br>2008-10 Thetford
Fixed-position photo-transects	2012	Moore & Thetford slope	2012-16 Moore<br>2012-16 Thetford
Fixed-position photo-transects	2018	Moore & Thetford slope	2018-20 Moore<br>2018-21 Thetford
Manta tow	2008	Thetford	2008-10 Thetford<br>2008-10 Thetford Front<br>2008-10 Thetford Back<br>2008-10 Thetford Flank1<br>2008-10 Thetford Flank2
Manta tow	2012	Moore & Thetford	2012-16 Thetford<br>2012-16 Thetford Front<br>2012-16 Thetford Back<br>2012-16 Thetford Flank1<br>2012-16 Thetford Flank2<br>2012-16 Moore<br>2012-16 Moore Front<br>2012-16 Moore Back<br>2012-16 Moore Flank1<br>2012-16 Moore Flank2
Manta tow	2018	Moore & Thetford	2018-22 Thetford<br>2018-22 Thetford Front<br>2018-22 Thetford Back<br>2018-22 Thetford Flank1<br>2018-22 Thetford Flank2<br>2018-21 Moore<br>2018-21 Moore Front<br>2018-21 Moore Back<br>2018-21 Moore Flank1<br>2018-21 Moore Flank2

4.2. Initialisation

As detailed in main text, simulations were initialised based on coral cover recorded in the AIMS long-term monitoring observations from the starting year of the hindcast (2008 for the full trajectory, or the start of each 'recovery window' for the population growth analysis). For the manta tow evaluation, coral cover was based on the manta tow observations, while the initial composition of the corymbose *Acropora* to the small sub-massive *Goniastrea* group was based on the ratio observed in the phototransect dataset at the initialisation year. Any site polygons directly underlying the sites of the phototransects or the manta tows were assigned the coral cover and composition values matching to the phototransect/tows. Initialisation for sites which did not underly a phototransect or tow was extrapolated. This was done by calculating a ratio (KR) between the coral habitat assigned to a site (K_i) and the initialisation

total coral cover T_{cover_i} determined for that site from the overlapping LTMP data, i.e. $KR_i = \frac{T_{cover\_site_i}}{K_i}$.

This allowed us to form a normal distribution N(μ=mean(KR), σ =sd(KR)). To extrapolate initial total coral cover for the remaining non-overlapping sites, we drew a sample KR from this distribution and calculated the coral cover:
$T_{cover\_site_i} = KR_i \times K_i$

5. Description of inputs used for simulations

Table S 5. Summary of inputs, resources and a priori modelling required to run C~scape simulations in the present study.

Model input	Description	Components used to generate model input	References / Source
Spatial file	Delineates sites and characterises them according to physical and environmental factors (e.g. depth, coral habitat suitability). The spatial file is generated in R.	Geomorphic zone map: Publicly available resource (.tif file) depicting the distribution of geomorphic features such as reef crest, slopes, reef flats and lagoons on a 10x10m grid. Used to delineate sites.	Roelfsema *et al.* (2021)
		Benthic habitat map: Publicly available resource (.tif file) classifying 10x10m pixels across reefs as one of four categories (Sand, Rubble, Rock, Coral/Algae). Used to inform the calculation of coral habitat.	Roelfsema *et al.* (2021)
		Bathymetric map: Publicly available resource (.tif file) in 10x10m grid given depth. Used to assign depth to C~scape sites.	https://www.eomap.com/
Temporal file	Describes the agents of mortality at each site in each year.	Cyclone hindcast: Cyclone category (0-5) for each reef in each year.	Determined from information in the AIMS long-term monitoring of case study reefs (Emslie *et al.* 2020)
		Annual Max Degree Heating Weeks hindcast for each reef in each year.	See methodology for generating in Bozec *et al.* (2022)
		Downscaling of Max Degree Heating Weeks to site level using RECOM modelling from historical heatwave years.	Using RECOM: (Herzfeld 2009; Steven et al. 2019). See section S3.1.
		Annual total larvae arriving to each reef from reefs external to the C~scape model domain.	Simulated outputs from GBR-wide model *ReefMod-GBR* with the same environmental forcings (Bozec et al. 2022)
		Annual density of crown-of-thorns starfish arriving to each reef.	Simulated outputs from GBR-wide model *ReefMod-GBR* with the same environmental forcings (Bozec et al. 2022)
Connectivity file	A 2D matrix describing the probability of larvae produced at each site travelling to every other site.	Hydrodynamic model: eReefs RECOM model nested within GBR1 was used to simulate hydrodynamics with a ~250 m spatial resolution.	RECOM: (Herzfeld 2009; Steven et al. 2019)<br>GBR1: (Skerratt et al. 2023)
		Tracer release: Coral larvae dispersal was simulated using passive tracers released at times that reflected the timing of annual mass spawning events for the two coral types.	See Main text
Integral Projection Model	Describes how a given coral population of known abundance and sizes will change over an annual period	Growth and survival data of coral colonies: Bayesian statistical regressions fit as a function of coral size in R using the brms package.	See section S2.3-2.5
		Early life history parameters: Probability of transitioning from a coral egg to a larvae, from a larvae to a settler, and from a settler to a small coral.	See Table S1, section S2.6
Initialisation information	Specify how to start simulations	Coral cover in year of initialisation was based on the manta tow observations, while the initial composition of the two coral types was based on the ratio observed in the phototransect dataset at the initialisation year.	See section S4.2, (Emslie *et al.* 2020)

References

Álvarez-Noriega, M., Baird, A.H., Dornelas, M., Madin, J.S., Cumbo, V.R. & Connolly, S.R. (2016) Fecundity and the demographic strategies of coral morphologies. *Ecology,* **97,** 3485-3493.

Babcock, R.C. (1991) Comparative demography of three species of scleractinian corals using age-and size-dependent classifications. *Ecological Monographs,* **61,** 225-244.

Baird, A.H., Madin, J.S., Álvarez-Noriega, M., Fontoura, L., Kerry, J.T., Kuo, C.-Y., Precoda, K., Torres-Pulliza, D., Woods, R.M. & Zawada, K.J. (2018) A decline in bleaching suggests that depth can provide a refuge from global warming in most coral taxa. *Marine Ecology Progress Series,* **603,** 257-264.

Bozec, Y.-M., Hock, K., Mason, R.A.B., Baird, M.E., Castro-Sanguino, C., Condie, S.A., Puotinen, M., Thompson, A. & Mumby, P.J. (2022) Cumulative impacts across Australia’s Great Barrier Reef: a mechanistic evaluation. *Ecological Monographs,* **92,** e01494.

Bürkner, P.-C. (2017) brms: An R package for Bayesian multilevel models using Stan. *Journal of Statistical Software,* **80,** 1-28.

Caswell, H. (2000) *Matrix population models*. Sinauer Sunderland, MA.

Cruz, D.W.d. & Harrison, P.L. (2017) Enhanced larval supply and recruitment can replenish reef corals on degraded reefs. *Scientific Reports,* **7,** 13985.

De'ath, G. & Moran, P. (1998) Factors affecting the behaviour of crown-of-thorns starfish (Acanthaster planci L.) on the Great Barrier Reef:: 2: Feeding preferences. *Journal of Experimental Marine Biology and Ecology,* **220,** 107-126.

Dela Cruz, D.W. & Harrison, P.L. (2020) Enhancing coral recruitment through assisted mass settlement of cultured coral larvae. *PLoS ONE,* **15,** e0242847.

Doropoulos, C., Elzinga, J., ter Hofstede, R., van Koningsveld, M. & Babcock, R.C. (2019) Optimizing industrial-scale coral reef restoration: comparing harvesting wild coral spawn slicks and transplanting gravid adult colonies. *Restoration Ecology,* **27,** 758-767.

Doropoulos, C., Ward, S., Roff, G., González-Rivero, M. & Mumby, P.J. (2015) Linking demographic processes of juvenile corals to benthic recovery trajectories in two common reef habitats. *PLoS ONE,* **10,** e0128535.

Elderd, B.D. & Miller, T.E. (2016) Quantifying demographic uncertainty: Bayesian methods for integral projection models. *Ecological Monographs,* **86,** 125-144.

Emslie, M.J., Bray, P., Cheal, A.J., Johns, K.A., Osborne, K., Sinclair-Taylor, T. & Thompson, C.A. (2020) Decades of monitoring have informed the stewardship and ecological understanding of Australia's Great Barrier Reef. *Biological Conservation,* **252,** 108854.

Engelhardt, U., Hartcher, M., Taylor, N., Cruise, J., Engelhardt, D., Russell, M., Stevens, I., Thomas, G., Williams, D. & Wiseman, D. (2001) *Crown-of-thorns Starfish (Acanthaster Planci) in the Central Great Barrier Reef Region: Results of Fine-scale Surveys Conducted in 1999-2000*. CRC Reef Research Centre Australia.

Fabricius, K.E., De'Ath, G., Puotinen, M.L., Done, T., Cooper, T.F. & Burgess, S.C. (2008) Disturbance gradients on inshore and offshore coral reefs caused by a severe tropical cyclone. *Limnology and oceanography,* **53,** 690-704.

Foster, T. & Gilmour, J. (2020) Egg size and fecundity of biannually spawning corals at Scott Reef. *Scientific Reports,* **10,** 1-9.

Hansen, P.E. (1989) Leslie matrix models. *Mathematical Population Studies,* **2,** 37-67.

Harrison, P.L., dela Cruz, D.W., Cameron, K.A. & Cabaitan, P.C. (2021) Increased coral larval supply enhances recruitment for coral and fish habitat restoration. *Frontiers in Marine Science,* **8,** 750210.

Herzfeld, M. (2009) Improving stability of regional numerical ocean models. *Ocean Dynamics,* **59,** 21-46.

Hughes, T.P. & Jackson, J. (1985) Population dynamics and life histories of foliaceous corals. *Ecological Monographs,* **55,** 141-166.

Hughes, T.P., Kerry, J.T., Baird, A.H., Connolly, S.R., Dietzel, A., Eakin, C.M., Heron, S.F., Hoey, A.S., Hoogenboom, M.O., Liu, G., McWilliam, M.J., Pears, R.J., Pratchett, M.S., Skirving, W.J., Stella, J.S. & Torda, G. (2018) Global warming transforms coral reef assemblages. *Nature,* **556,** 492-496.

Kayal, M., Lenihan, H.S., Brooks, A.J., Holbrook, S.J., Schmitt, R.J. & Kendall, B.E. (2018) Predicting coral community recovery using multi-species population dynamics models. *Ecology Letters,* **21,** 1790-1799.

Keesing, J.K. & Lucas, J.S. (1992) Field measurement of feeding and movement rates of the crown-of-thorns starfish Acanthaster planci (L.). *Journal of Experimental Marine Biology and Ecology,* **156,** 89-104.

Kennedy, E.V., Roelfsema, C.M., Lyons, M.B., Kovacs, E.M., Borrego-Acevedo, R., Roe, M., Phinn, S.R., Larsen, K., Murray, N.J. & Yuwono, D. (2021) Reef Cover, a coral reef classification for global habitat mapping from remote sensing. *Scientific Data,* **8,** 196.

Lyons, M.B., Roelfsema, C.M., Kennedy, E.V., Kovacs, E.M., Borrego-Acevedo, R., Markey, K., Roe, M., Yuwono, D.M., Harris, D.L., Phinn, S.R., Asner, G.P., Li, J., Knapp, D.E., Fabina, N.S., Larsen, K., Traganos, D. & Murray, N.J. (2020) Mapping the world's coral reefs using a global multiscale earth observation framework. *Remote Sensing in Ecology and Conservation,* **6,** 557-568.

Merow, C., Dahlgren, J.P., Metcalf, C.J.E., Childs, D.Z., Evans, M.E.K., Jongejans, E., Record, S., Rees, M., Salguero-Gómez, R. & McMahon, S.M. (2014) Advancing population ecology with integral projection models: a practical guide. *Methods in Ecology and Evolution,* **5,** 99-110.

Metcalf, C.J.E., McMahon, S.M., Salguero-Gómez, R. & Jongejans, E. (2013) IPMpack: an R package for integral projection models. *Methods in Ecology and Evolution,* **4,** 195-200.

Roelfsema, C.M., Kovacs, E.M., Ortiz, J.C., Callaghan, D.P., Hock, K., Mongin, M., Johansen, K., Mumby, P.J., Wettle, M., Ronan, M., Lundgren, P., Kennedy, E.V. & Phinn, S.R. (2020) Habitat maps to enhance monitoring and management of the Great Barrier Reef. *Coral Reefs,* **39,** 1039-1054.

Roelfsema, C.M., Lyons, M.B., Castro-Sanguino, C., Kovacs, E.M., Callaghan, D., Wettle, M., Markey, K., Borrego-Acevedo, R., Tudman, P., Roe, M., Kennedy, E.V., Gonzalez-Rivero, M., Murray, N. & Phinn, S.R. (2021) How Much Shallow Coral Habitat Is There on the Great Barrier Reef? *Remote Sensing,* **13,** 4343.

Skerratt, J., Baird, M.E., Mongin, M., Ellis, R., Smith, R.A., Shaw, M. & Steven, A.D.L. (2023) Dispersal of the pesticide diuron in the Great Barrier Reef. *Science of the Total Environment,* **879,** 163041.

Steven, A.D.L., Baird, M.E., Brinkman, R., Car, N.J., Cox, S.J., Herzfeld, M., Hodge, J., Jones, E., King, E., Margvelashvili, N., Robillot, C., Robson, B., Schroeder, T., Skerratt, J., Tickell, S., Tuteja, N., Wild-Allen, K. & Yu, J. (2019) eReefs: An operational information system for managing the Great Barrier Reef. *Journal of Operational Oceanography,* **12,** S12-S28.

Wallace, C. (1985) Seasonal peaks and annual fluctuations in recruitment of juvenile scleractinian corals. *Marine Ecology Progress Series***,** 289-298.
Wijffels, S.E., Beggs, H., Griffin, C., Middleton, J.F., Cahill, M., King, E., Jones, E., Feng, M., Benthuysen, J.A. & Steinberg, C.R. (2018) A fine spatial-scale sea surface temperature atlas of the Australian regional seas (SSTAARS): Seasonal variability and trends around Australasia and New Zealand revisited. *Journal of Marine Systems,* **187,** 156-196.
